# Multiple molecular mechanisms form a positive feedback loop driving amyloid β42 peptide-induced neurotoxicity via activation of the TRPM2 channel in hippocampal neurons

**DOI:** 10.1038/s41419-018-0270-1

**Published:** 2018-02-07

**Authors:** Xin Li, Lin-Hua Jiang

**Affiliations:** 1 School of Biomedical Sciences, Faculty of Biological Sciences, University of Leeds, Leeds, UK; 2 Sino-UK Joint Laboratory of Brain Function and Injury of Henan Province and Department of Physiology and Neurobiology, Xinxiang Medical University, Xinxiang, China

## Abstract

Emerging evidence supports an important role for the ROS-sensitive TRPM2 channel in mediating age-related cognitive impairment in Alzheimer’s disease (AD), particularly neurotoxicity resulting from generation of excessive neurotoxic Aβ peptides. Here we examined the elusive mechanisms by which Aβ_42_ activates the TRPM2 channel to induce neurotoxicity in mouse hippocampal neurons. Aβ_42_-induced neurotoxicity was ablated by genetic knockout (TRPM2-KO) and attenuated by inhibition of the TRPM2 channel activity or activation through PARP-1. Aβ_42_-induced neurotoxicity was also inhibited by treatment with TPEN used as a Zn^2+^-specific chelator. Cell imaging revealed that Aβ_42_-induced lysosomal dysfunction, cytosolic Zn^2+^ increase, mitochondrial Zn^2+^ accumulation, loss of mitochondrial function, and mitochondrial generation of ROS. These effects were suppressed by TRPM2-KO, inhibition of TRPM2 or PARP-1, or treatment with TPEN. Bafilomycin-induced lysosomal dysfunction also resulted in TRPM2-dependent cytosolic Zn^2+^ increase, mitochondrial Zn^2+^ accumulation, and mitochondrial generation of ROS, supporting that lysosomal dysfunction and accompanying Zn^2+^ release trigger mitochondrial Zn^2+^ accumulation and generation of ROS. Aβ_42_-induced effects on lysosomal and mitochondrial functions besides neurotoxicity were also suppressed by inhibition of PKC and NOX. Furthermore, Aβ_42_-induced neurotoxicity was prevented by inhibition of MEK/ERK. Therefore, our study reveals multiple molecular mechanisms, including PKC/NOX-mediated generation of ROS, activation of MEK/ERK and PARP-1, lysosomal dysfunction and Zn^2+^ release, mitochondrial Zn^2+^ accumulation, loss of mitochondrial function, and mitochondrial generation of ROS, are critically engaged in forming a positive feedback loop that drives Aβ_42_-induced activation of the TRPM2 channel and neurotoxicity in hippocampal neurons. These findings shed novel and mechanistic insights into AD pathogenesis.

## Introduction

Alzheimer’s disease (AD) is an age-related neurodegenerative disorder characterized by progressive cognitive impairments and representing the most prevalent cause of dementia among the elder people. One histopathological hallmark of AD is the formation of senile amyloid plaque with deposits of amyloid β (Aβ) peptides resulting from proteolytic cleavage of amyloid precursor protein (APP) by presenilin-1 (PS-1) containing γ-secretase^1^. It is known that Aβ induce neurotoxicity via multiple but yet not fully understood mechanisms, leading to synaptic loss and network dysfunction in hippocampus and other brain regions^2^. For example, Aβ can stimulate generation of reactive oxygen species (ROS) in hippocampal neurons^3^. In addition, lipid peroxides and oxidative modifications of proteins and lipids are widely observed in cells exposed to Aβ and in the brain of transgenic APP/PS-1 AD mice, consistent with a role for oxidative stress in Aβ-induced neurotoxicity^4,5^. Zn^2+^, as one of the most common trace elements in human body, has numerous structural and regulatory functions, but it is highly neurotoxic^6,7^. Zn^2+^ can enhance oxidative stress via impairing mitochondrial function and inducing mitochondrial generation of ROS or activating other ROS-generating mechanisms such as NADPH-dependent oxidases (NOX) ^8,9^. In fact, NOX are an important source of ROS that induce neuronal death implicated in ischemic stroke and AD^10,11^. Conversely, oxidative stress can elevate the cytosolic Zn^2+^ concentration ([Zn^2+^]_c_) by activating diverse Ca^2+^/Zn^2+^-transporting mechanisms that mediate extracellular Zn^2+^ influx and/or Zn^2+^ release from intracellular organelles such as lysosome, or inducing Zn^2+^ release from cytosolic Zn^2+^-binding metallothioneins^6,7,12–15^. Such intimate relationships of ROS and Zn^2+^ in neurotoxicity are well-documented under ischemic stroke but less understood in AD, particularly Aβ-induced neurotoxicity.

Transient receptor potential melastatin-related 2 (TRPM2) is a Ca^2+^-permeable channel primarily located on cell surface^16,17^ and also function as a lysosomal Ca^2+^-release channel in pancreatic β-cells and dendritic cells^18,19^. TRPM2 channel is gated by intracellular ADP-ribose (ADPR), and potently activated by ROS, mainly via stimulating ADPR-generating mechanisms^20,21^, and confers susceptibility to ROS-induced cell death^22^ in numerous cell types^20,23^. For example, TRPM2 channel mediates neuronal death in vitro induced by H_2_O_2_ and ROS-inducing stimuli including Aβ_42_, or under in vivo conditions known to promote generation of ROS such as ischemic stroke^24–31^. Consistently with an early in vitro study suggesting a role for the TRPM2 channel in Aβ_42_-induced neurotoxicity^24^, a recent study shows that genetic ablation of TRPM2 in the APP/PS-1 mice prevented neurotoxicity and age-related memory impairment^32^, supporting a causative relationship of the TRPM2 channel with AD, particularly Aβ-induced neurotoxicity and cognitive dysfunction. However, it remained elusive how Aβ activate the TRPM2 channel to induce neurotoxicity. Our recent study shows an exclusive role for the TRPM2 channel in elevating the [Zn^2+^]_c_ that is critical in post-ischemia hippocampal neuronal death and impaired learning and memory^30^. In this study, we aimed to elucidate the mechanisms for Aβ_42_-induced TRPM2 channel activation, alteration in intracellular Zn^2+^ homeostasis and neurotoxicity in hippocampal neurons.

## Results

### TRPM2 in Aβ_42_-induced hippocampal neurotoxicity

**Fig. 1:**
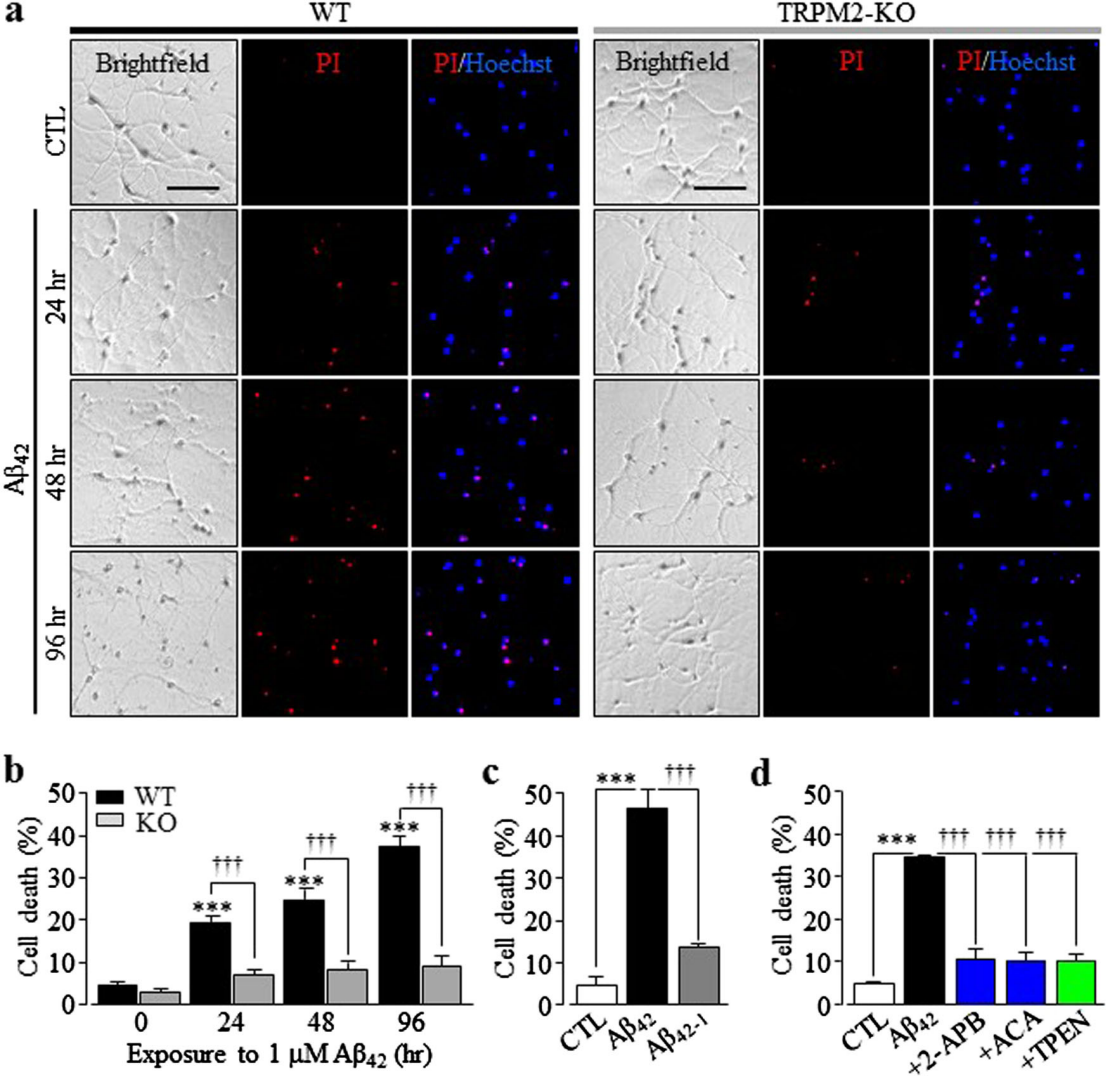
Aβ_42_ induces TRPM2-dependent and Zn^2+^-dependent hippocampal neuronal death. **a** Representative images showing PI staining of wild-type (WT) and TRPM2-KO neurons under control (CTL) conditions or after exposure to 1 µM Aβ_42_ for 24, 48, or 96 h. Each panel consists of brightfield image showing neurons, PI-staining image (red) showing dead neurons and merged Hoechst (blue)/PI-staining image showing all and dead neurons. Scale bar is 100 µm. **b** Summary of the mean percentage of PI-positive neurons under indicated conditions from 3–5 independent experiments, with each experiment examining 400–650 neurons for each condition. Black and gray bars represent the percentage of neuronal death in WT and TRPM2-KO neurons, respectively. ***p < 0.005 indicates significant difference from respective CTL. ^†††^*p* < 0.005 indicates significant difference between WT and TRPM2-KO neurons. **c** Summary of the mean percentage of PI-positive neurons or cell death in WT neurons after exposure to 1 μM Aβ_42_ or control peptide Aβ_42-1_ for 96 h, from three independent experiments, with each experiment examining 350–500 neurons. ***p < 0.005 indicates significant difference from CTL. ^†††^*p* < 0.005 indicates significant difference between treatments with two different peptides. **d** Summary of the mean percentage of PI-positive neurons in WT neurons after exposure to 1 µM Aβ_42_ for 96 h, without or with treatment with 10 µM 2-APB, 1 μM ACA, or 100 nM TPEN, 30 min prior to and during exposure to Aβ_42_, from three to five independent experiments, with each experiment examining 400–600 neurons for each condition. ***p < 0.005 indicates significant difference from CTL. ^†††^*p* < 0.005 indicates difference from neurons exposed with Aβ_42_ alone

To investigate TRPM2 in mediating Aβ-induced neurotoxicity, we started with PI-staining assay to determine hippocampal neuronal death induced by Aβ_42_, the major neurotoxic Aβ^33^. Exposure to Aβ_42_ at 100 and 300 ng/ml (~22 and 66 nM) for 24–96 h led to significant neuronal death in wild-type (WT) neurons (supplementary Fig.1). Exposure to 1 µM Aβ_42_ resulted in greater neuronal death (Fig. 1a,b) and, by contrast, exposure to 1 µM Aβ_42-1_, the peptide with a reversal sequence, caused minimal neuronal death (Fig. 1c; supplementary Fig.2). Aβ_42_-induced neurotoxicity was not observed in TRPM2-knockout (TRPM2-KO) neurons (Fig. 1a,b; supplementary Fig.1). Treatment of WT neurons with 10 μM 2-APB or 1 μM ACA, two TRPM2 channel inhibitors^20^, strongly suppressed Aβ_42_-induced neurotoxicity (Fig. 1d). These results provide genetic and pharmacological evidence to demonstrate a critical role for the TRPM2 channel in neurotoxicity induced by Aβ_42_ at biologically relevant concentrations, in agreement with a recent study showing prevention by TRPM2-KO of hippocampal neurotoxicity due to excessive Aβ generation in the APP/PS-1 mice^32^. Treatment with 1 μM PJ34 or 30 μM DPQ, two poly(ADPR) polymerase-1 (PARP-1) inhibitors, also significantly attenuated Aβ_42_-induced neurotoxicity (supplementary Fig.3), consistent with engagement of PARP-1 in Aβ_42_-induced TRPM2 channel activation and neurotoxicity, as previously suggested^24^.

### TRPM2 in Aβ_42_-induced increase in the [Zn^2+^]_c_ and lysosome dysfunction

**Fig. 2:**
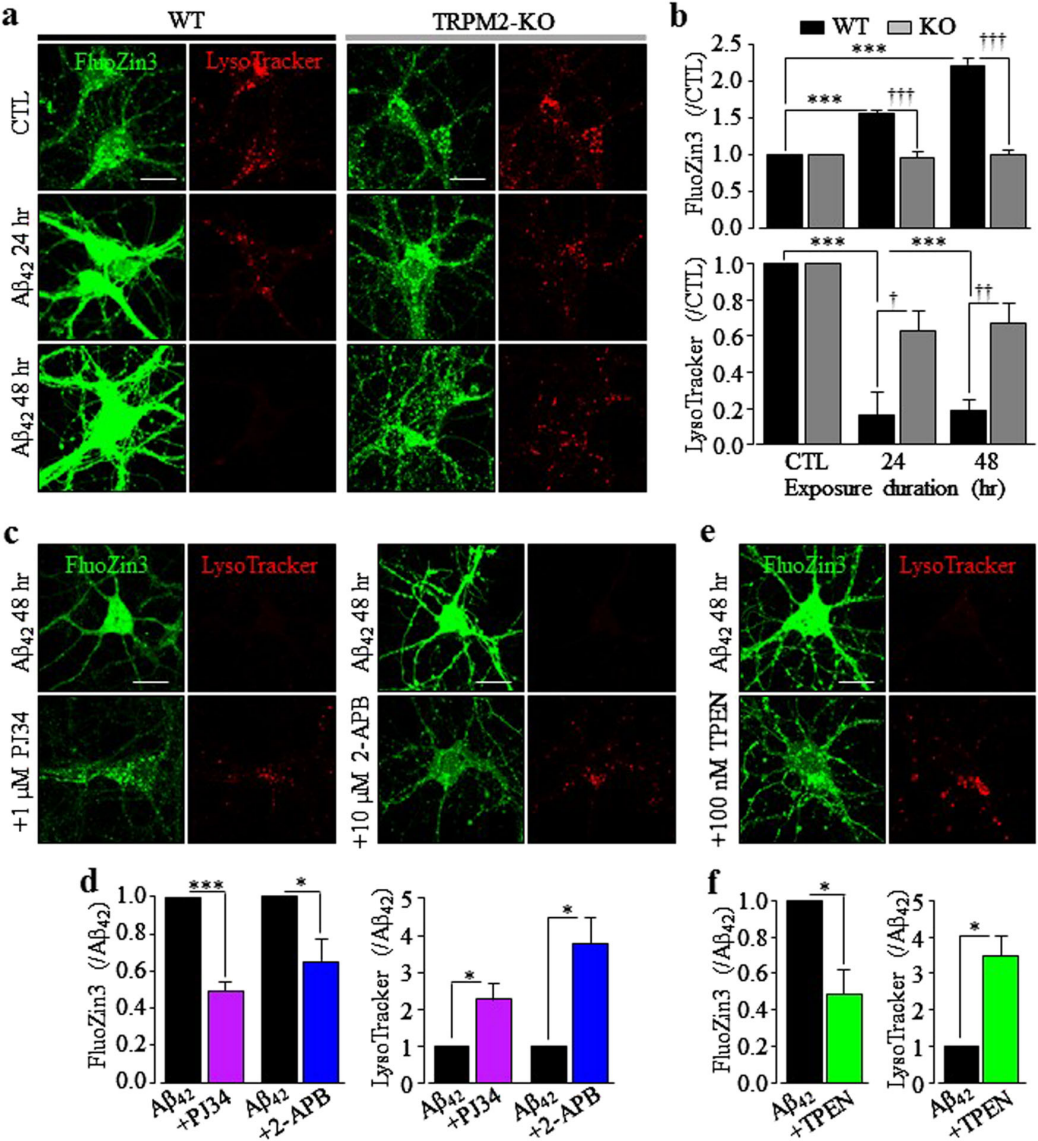
Aβ_42_ induces TRPM2-dependent increase in the [Zn^2+^]_c_ and lysosomal dysfunction in hippocampal neurons. **a**, **c**, **e** Representative confocal images showing FluoZin3 (green) and LysoTracker (red) staining of wild-type (WT) and TRPM2-KO neurons under control (CTL) conditions or after exposure to 1 µM Aβ_42_ for 24 and 48 h **a**, WT neurons after exposure to 1 µM Aβ_42_ for 48 h with or without treatment with 1 µM PJ34, 10 µM 2-APB **c**, or 100 nM TPEN **e**, 30 min prior to and during exposure to Aβ_42_. Scale bar is 10 µm. **b**, **d**, **f** Summary of the mean fluorescence intensity of FluoZin3 (top panel in **b**, and left panel in **d** and **f**) or LysoTracker (bottom panel in **b**, and right panel in **d** and **f**) under indicated conditions, from three to four independent experiments, with each experiment examining 10–15 neurons for each condition. *p < 0.05; and ***p < 0.005 indicate significant difference from CTL **b** or neurons treated with Aβ_42_ alone (**d**, **f**). ^†^*p* < 0.05; ^††^*p* < 0.01; and ^†††^*p* < 0.005 indicate significant difference between WT and TRPM2-KO neurons **b**

We recently show a critical role of TRPM2-dependent increase in the [Zn^2+^]_c_ in post-ischemia hippocampal neuronal death^30^. Therefore, we examined whether TRPM2-dependent alteration in intracellular Zn^2+^ homeostasis is important in Aβ_42_-induced neurotoxicity. Treatment with 100 nM TPEN, as a selective Zn^2+^ chelator^34^, almost completely prevented Aβ_42_-induced neurotoxicity (Fig. 1d), indicating a vital role of a rising [Zn^2+^]_c_ in Aβ_42_-induced neurotoxicity. We used single-cell imaging and Fluozin3, an indicator for labile Zn^2+^, to examine the [Zn^2+^]_c_ in individual hippocampal neurons. As reported previously^30^, the [Zn^2+^]_c_ in untreated neurons was low and Zn^2+^ was mainly concentrated in discrete puncta. Here, we further showed that such Zn^2+^ puncta exhibited strong co-localization with LysoTracker, but not with MitoTracker (supplementary Fig.4), indicating that Zn^2+^ is predominantly of lysosomal origin, as recently reported in pancreatic β-cells and endothelial cells^35,36^. Exposure of WT neurons to Aβ_42_ for 24–48 h induced a salient increase in the [Zn^2+^]_c_, and noticeable decline in LysoTracker intensity that suggests lysosomal dysfunction (Fig. 2a,b). However, Aβ_42_ induced no discernible change in the [Zn^2+^]_c_ or LysoTracker intensity in TRPM2-KO neurons (Fig. 2a,b). Aβ_42_-induced increase in the [Zn^2+^]_c_ and reduction in LysoTracker intensity were strongly suppressed by treatment with 1 μM PJ34 and 10 μM 2-APB (Fig. 2c,d) or 100 nM TPEN (Fig. 2e,f). Collectively, these results show that the TRPM2 channel is crucial for Aβ_42_-induced increase in the [Zn^2+^]_c_ and lysosomal dysfunction. To provide further evidence to demonstrate Aβ_42_-induced lysosomal dysfunction, we performed single-cell imaging using acridine orange (AO), a fluorescence indicator that emits red fluorescence when it is entrapped in lysosome. As a positive control, bafilomycin caused complete loss of AO fluorescence (supplementary Fig.5a). Exposure to Aβ_42_ for 96 h significantly reduced the AO intensity in WT but not TRPM2-KO neurons (supplementary Fig. 5a-c), supporting Aβ_42_-induced TRPM2-dependent lysosomal dysfunction.

### TRPM2 in Aβ_42_-induced mitochondrial Zn^2+^ accumulation, loss of mitochondria function, change in mitochondrial morphology, and mitochondrial generation of ROS

**Fig. 3:**
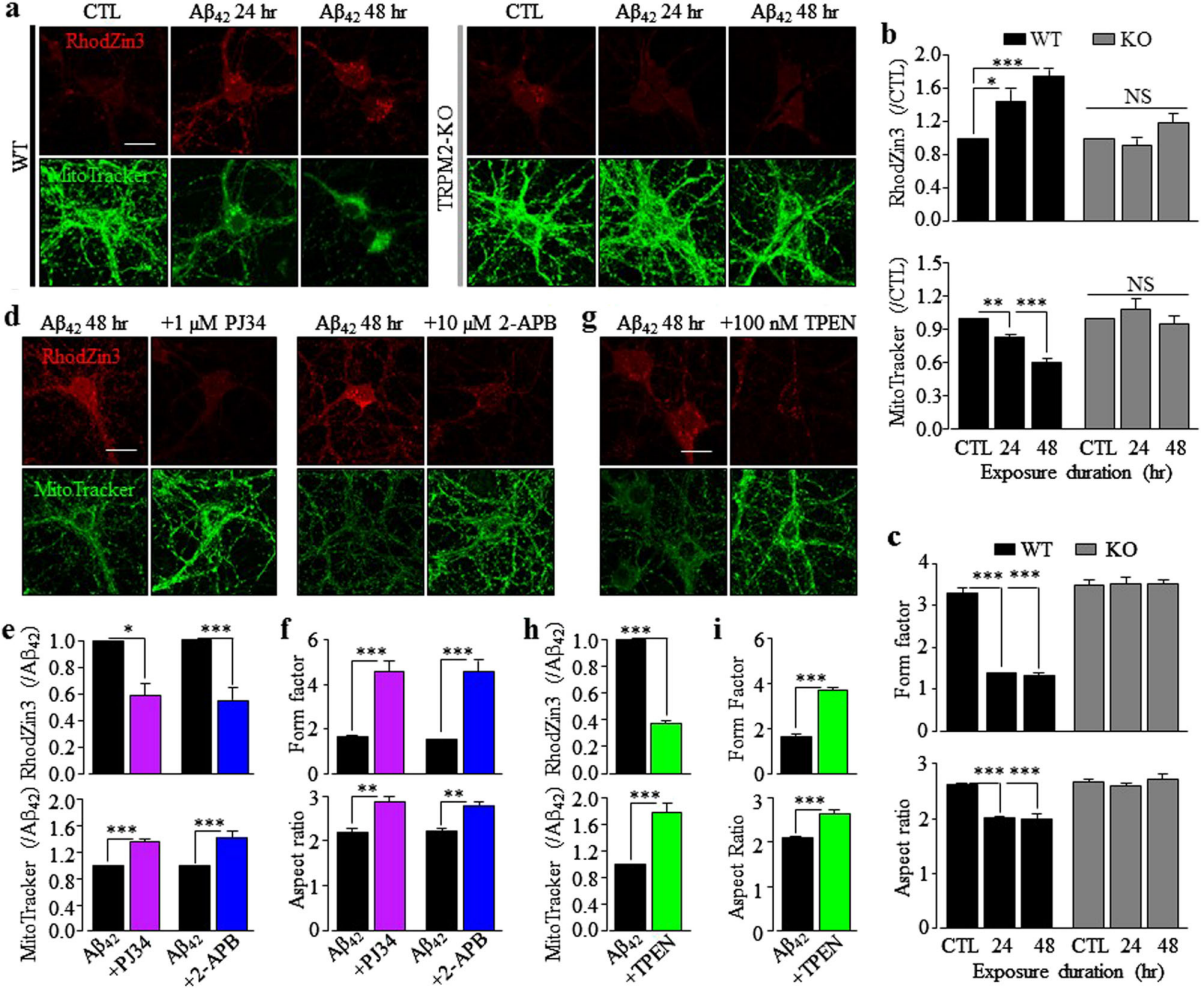
Aβ_42_ induces TRPM2-dependent mitochondrial Zn^2+^ accumulation, loss of mitochondrial function and change in mitochondrial morphology in hippocampal neurons. **a**, **d**, **g** Representative confocal images showing RhodZin3 (red) and MitoTracker (green) staining of wild-type (WT) and TRPM2-KO neurons under control (CTL) conditions or after exposure to 1 µM Aβ_42_ for 24 and 48 h **a**, WT hippocampal neurons after exposure to 1 µM Aβ_42_ for 48 h with or without treatment with 1 µM PJ34 or 10 µM 2-APB **d**, or 100 nM TPEN **g**, 30 min prior to and during exposure to Aβ_42_. Scale bar is 10 µm. **b**, **e**, **h** Summary of the mean fluorescence intensity of RhodZin3 (top panels) and MitoTracker (bottom panels) under indicated conditions, normalized to that in CTL neurons **b** or neurons treated with Aβ_42_ alone **e**, **h**. **c**, **f**, **i** Summary of the mean aspect ratio and form factor of mitochondria in neurons under indicated conditions. The data shown in **b**–**c**, **e**–**f**,** h**–**i** were from three to four independent experiments, with each examining 15–20 neurons for each condition. *p < 0.05 and ***p < 0.005 indicate significant difference from CTL **b**, **c** or neurons treated with Aβ_42_ alone (**e**–**f**, **h**–**i**). NS no significant difference

Increasing evidence shows loss of mitochondria or mitochondrial function in neurons in the close vicinity or contact with Aβ-laden senile plaque^5,37–39^. As introduced above, Zn^2+^ bears an intimate relationship with loss of mitochondrial function and mitochondrial generation of ROS. Therefore, we performed singe-cell imaging to examine mitochondrial Zn^2+^ accumulation using RhodZin3 and ensuing effects on the mitochondrial function using MitoTracker Green. Exposure of WT neurons to Aβ_42_ for 24–48 h stimulated substantial mitochondrial Zn^2+^ accumulation and also strong reduction in MitoTracker intensity that suggests loss of mitochondrial function (Fig. 3a,b). Consistently, there was a low but significant level of cytochrome-c (Cyt-c) release in WT neurons after exposure to Aβ_42_ for 24–48 h detected by immunostaining (supplementary Fig.6). Analysis of the form factor and aspect ratio of mitochondria reveals salient change in their morphology (Fig .3a,c, supplementary Fig.7 and supplementary Fig.8a). Aβ_42_-induced mitochondrial Zn^2+^ accumulation, loss of MitoTracker intensity and change in mitochondrial morphology were abolished by TRPM2-KO (Fig. 3a-c, supplementary Fig.7 and supplementary Fig.8b) and strongly inhibited by treatment with 1 μM PJ34 or 10 μM 2-APB (Fig. 3d-f; supplementary Fig.8c-d). Aβ_42_-induced mitochondrial effects in WT neurons were also suppressed by treatment with 100 nM TPEN (Fig. 3g-i; supplementary Fig.8e). Taken together, these results show that Aβ_42_-induced TRPM2-dependent mitochondrial Zn^2+^ accumulation causes loss of mitochondrial function and change in mitochondrial morphology.

**Fig. 4:**
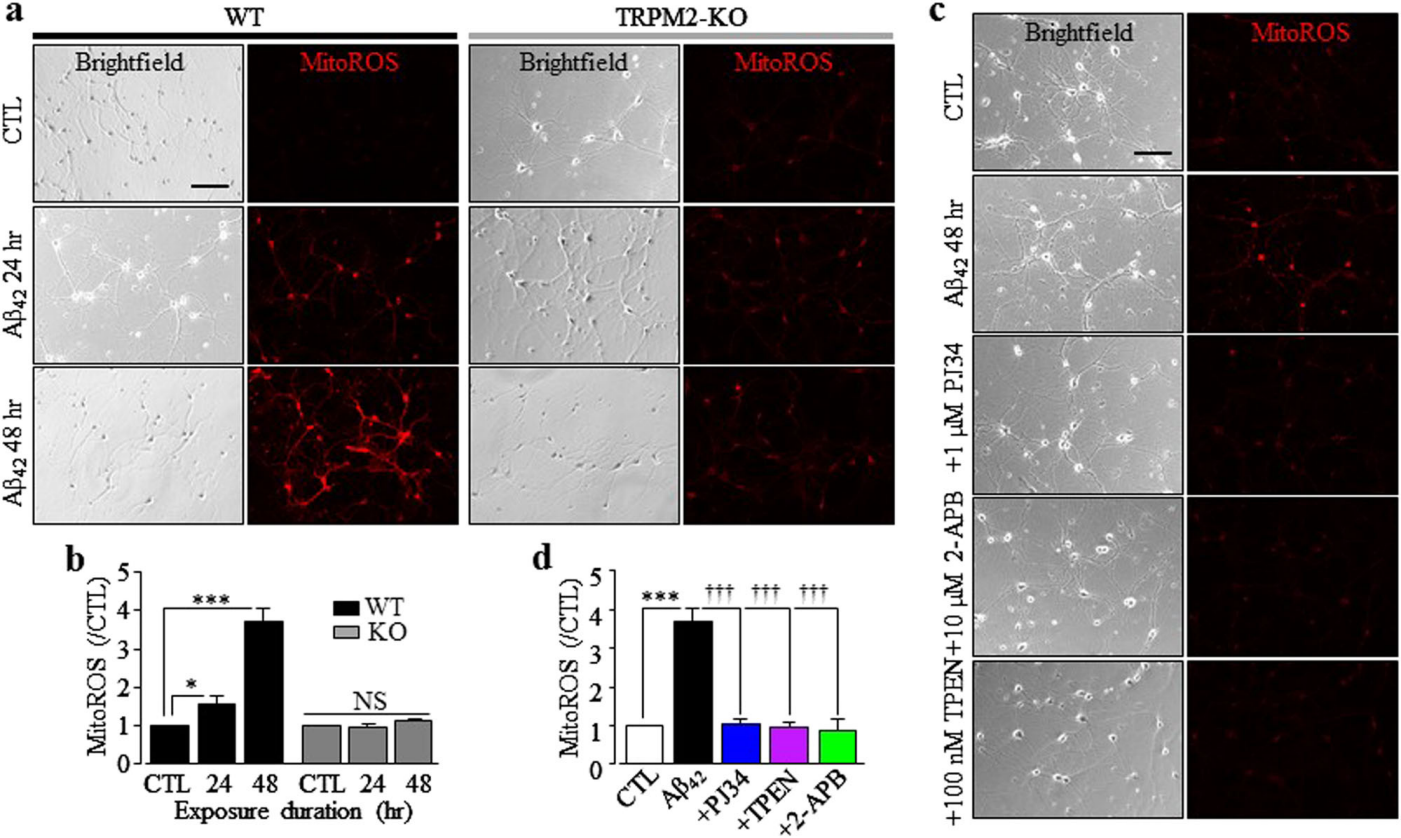
Aβ_42_-induced TRPM2-dependent mitochondrial Zn^2+^ accumulation triggers mitochondrial generation of ROS in hippocampal neurons. **a**, **c** Representative images showing MitoTracker Red CM-H_2_ros staining (MitoROS; red) of wild-type (WT) and TRPM2-KO neurons under control (CTL) conditions and after exposure to 1 µM Aβ_42_ for 24 and 48 h **a**, and WT neurons after exposure to 1 µM Aβ_42_ for 48 h with or without treatment with 1 µM PJ34, 10 µM 2-APB, or 100 nM TPEN, 30 min prior to and during exposure to Aβ_42_ **c**. Scale bar is 100 µm. **b**, **d** Summary of the mean MitoTracker Red CM-H_2_ros red fluorescence intensity (MitoROS) normalized to that in CTL neurons. The data were from three independent experiments, with each experiment examining 350–500 neurons for each condition. *p < 0.05; and ***p < 0.005 indicate difference from CTL. ^†††^*p* < 0.005 indicates difference from neurons exposed with Aβ_42_ alone. NS no significant difference

We next investigated whether Aβ_42_-induced mitochondrial Zn^2+^ accumulation stimulated generation of ROS and furthermore whether such generation of ROS was also dependent of TRPM2, using MitoTracker Red CM-H_2_Xros. The mitochondrial ROS level in WT neurons was increased by approximately 1.5-fold and 4-fold after exposure to Aβ_42_ for 24 and 48 h, respectively (Fig. 4a,b). In contrast, Aβ_42_ induced no mitochondrial generation of ROS in TRPM2-KO neurons (Fig. 4a,b) and in WT neurons treated with 1 μM PJ34 or 10 μM 2-APB (Fig. 4c,d). Similarly, there was no Aβ_42_-induced increase in mitochondrial generation of ROS in WT neurons treated with 100 nM TPEN (Fig. 4c,d). Therefore, these results clearly show that Aβ_42_-induced TRPM2-dependent mitochondrial Zn^2+^ accumulation stimulates mitochondrial generation of ROS.

### TRPM2 in bafilomycin-induced increase in the [Zn^2+^]_c_, mitochondrial Zn^2+^ accumulation, and mitochondrial generation of ROS

**Fig. 5:**
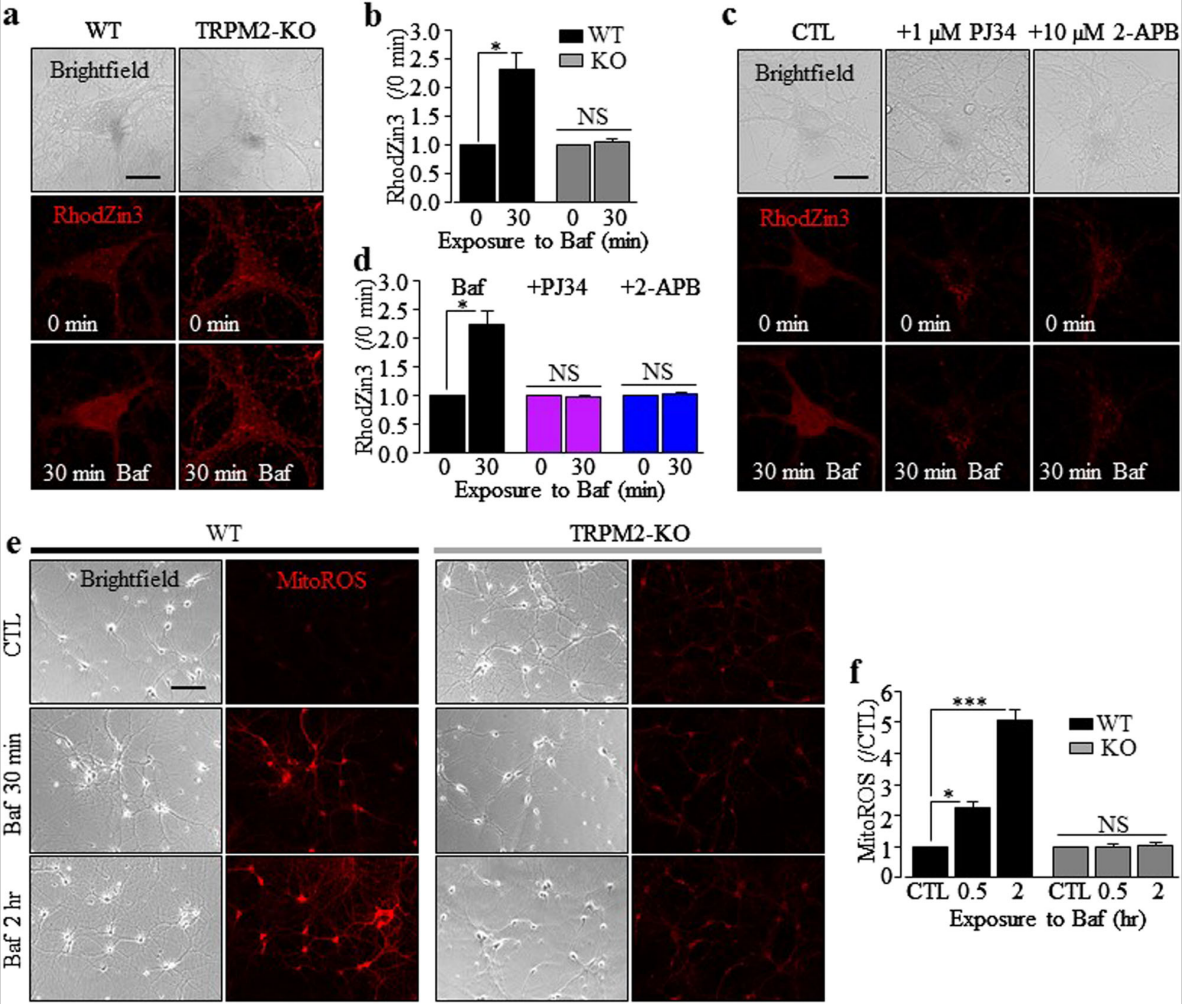
Bafilomycin induces TRPM2-dependent mitochondria Zn^2+^ accumulation and mitochondrial generation of ROS in hippocampal neurons. **a**, **c** Time-lapse confocal imaging of RhodZin3 fluorescence intensity during 30 min exposure to 100 nM bafilomycin (Baf) in wild-type (WT) and TRPM2-KO neurons **a**, or in WT neurons under control (CTL) conditions and after exposure to bafilomycin without or with treatment with 1 µM PJ34 or 10 µM 2-APB, 30 min prior to and during exposure to bafilomycin **c**. Scale bar is 10 µm. **b**, **d** Summary of the mean RhodZin3 fluorescence intensity under indicated conditions, normalized to the basal level (0 min), from three independent experiments, with each experiment examining 3–9 neurons for each condition. **e** Representative images showing MitoTracker Red CM-H_2_ros red fluorescence (MitoROS) in WT and TRPM2-KO neurons under CTL conditions or during exposure to 100 nM bafilomycin for 30 min and 2 h. Scale bar is 100 µm. **f** Summary of the mean MitoTracker Red CM-H_2_Xros red fluorescence intensity (MitoROS) under indicated conditions normalized to that in neurons under CTL conditions, from three to five independent experiments, with each experiment examining 40–70 neurons for each condition. *p < 0.05; and ***p < 0.005 indicate difference from the basal level **b**, **d** or CTL **f**; NS no significant difference

We hypothesized that Aβ_42_-induced lysosomal dysfunction triggers an increase in the [Zn^2+^]_c_ and subsequent mitochondrial Zn^2+^ accumulation. To seek supporting evidence, we returned to bafilomycin and asked whether bafilomycin-induced lysosomal dysfunction gave rise to an increase in the [Zn^2+^]_c_ and mitochondrial Zn^2+^ accumulation like Aβ_42_. Exposure to 100 nM bafilomycin for 30 min resulted in a significant increase in the [Zn^2+^]_c_ (supplementary Fig.9) and mitochondrial Zn^2+^ accumulation in WT neurons, which were prevented by TRPM2-KO (Fig. 5a,b; supplementary Fig.9). Bafilomycin-induced mitochondrial Zn^2+^ accumulation was also prevented by treatment with 1 μM PJ34 or 10 μM 2-APB (Fig. 5c,d). Furthermore, bafilomycin induced massive mitochondrial generation of ROS in WT neurons, but again not in TRPM2-KO neurons (Fig. 5e,f). These results, together with the above-described results from using Aβ_42_, strongly support the hypothesis that Aβ_42_-induced lysosomal dysfunction triggers an increase in the [Zn^2+^]_c_ and subsequent mitochondrial Zn^2+^ accumulation and mitochondrial generation of ROS in a TRPM2-dependent manner.

**Fig. 6:**
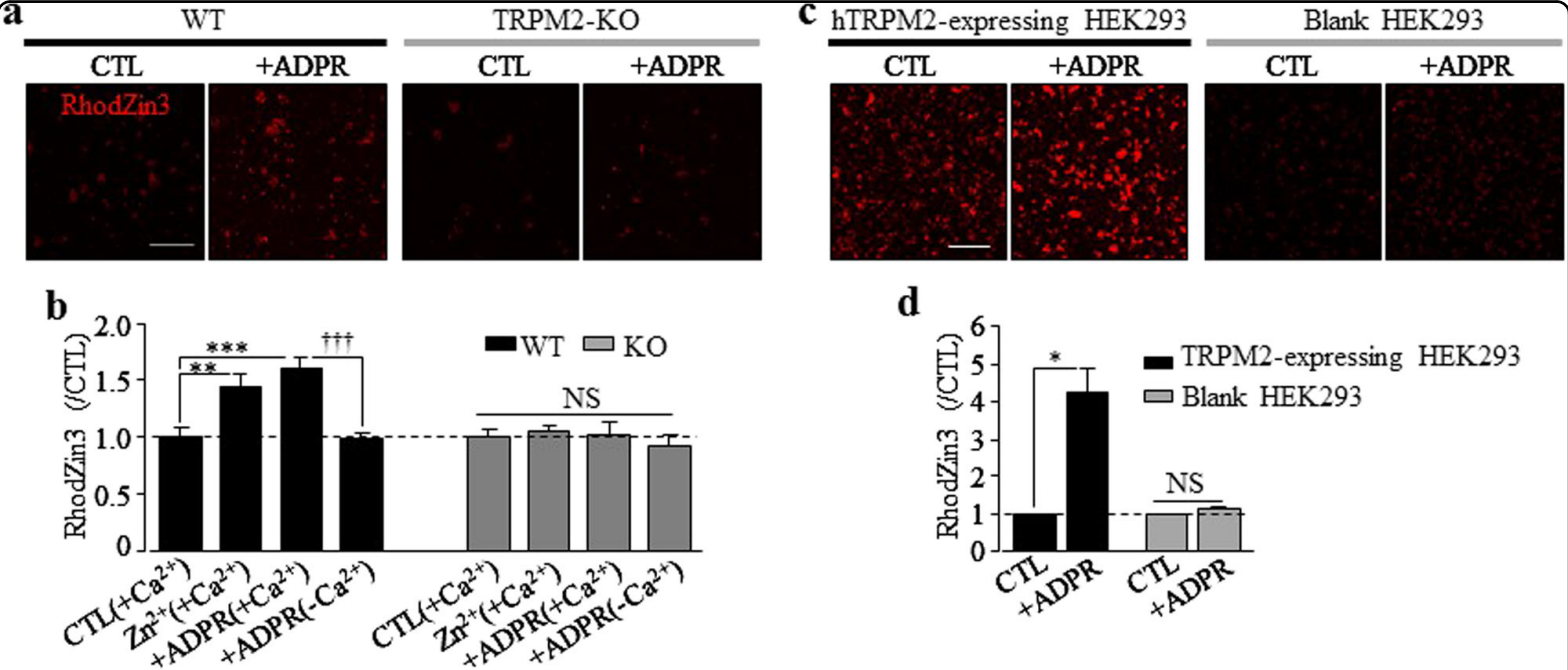
ADPR induces TRPM2-dependent Zn^2+^ accumulation in isolated mitochondria. **a** Representative images showing RhodZin3 staining of isolated mitochondria from wild-type (WT) and TRPM2-KO hippocampal neurons under control (CTL) condition or treatment with 5 mM ADPR, prior to addition of 30 µM Zn^2+^, in the extracellular solution containing 1.5 mM Ca^2+^. **b** Summary of the mean RhodZin3 fluorescence intensity in isolated mitochondria under indicated conditions (+Ca^2+^: Ca^2+^-containing solution; −Ca^2+^: Ca^2+^-free solution) normalized to the basal or CTL level, from 5 independent preparations. **c** Representative images showing RhodZin3 staining of mitochondria isolated from HEK293 cells overexpressing human TRPM2 channel (hTRPM2-expressing HEK293) or blank HEK293 cells without (CTL) or with treatment with 1 mM ADPR prior to addition of 30 µM Zn^2+^ in extracellular Ca^2+^-containing solution. **d** Summary of the mean RhodZin3 fluorescence intensity in mitochondria from HEK293 cells under indicated conditions normalized to that under CTL conditions, from three independent preparations. *p < 0.05; **p < 0.01; and ***p < 0.005 indicate difference from CTL. ^†††^*p* < 0.005 indicates difference in the presence and absence of Ca^2+^ in extracellular solutions; NS, no significant difference. Scale bar is 20 µm

The strong dependence on TRPM2 of bafilomycin/Aβ_42_-induced mitochondrial Zn^2+^ accumulation and mitochondrial generation of ROS raised an intriguing question regarding the TRPM2 channel in mitochondria. Immunostaining suggests that TRPM2 protein was present intracellularly in hippocampal neurons and exhibited co-localization with MitoTracker (supplementary Fig.10a-b). Western blotting also detected TRPM2 protein in mitochondria isolated from hippocampal neurons (supplementary Fig.10c). To further demonstrate the relevance of mitochondrial expression of TRPM2 to Zn^2+^ accumulation, we performed RhodZin3 imaging to monitor Zn^2+^ influx into isolated mitochondria from WT and TRPM2-KO hippocampal neurons. Addition of Zn^2+^ in the presence of Ca^2+^ led to a significant increase in the Zn^2+^ level in mitochondria isolated from WT neurons, which was further elevated by addition of ADPR, the TRPM2 channel specific activator (Fig. 6a,b). In contrast, ADPR induced no increase in the mitochondrial Zn^2+^ level in the absence of Ca^2+^ (Fig. 6b). Furthermore, such Zn^2+^ increases were not observed in mitochondria isolated from TRPM2-KO neurons (Fig. 6a,b). We also examined mitochondria isolated from blank HEK293 cells and HEK293 cells overexpressing the human TRPM2 channel (hTRPM2-expressing HEK293 cells). Western blotting showed a high level of TRPM2 protein in mitochondria from hTRPM2-expressing HEK293 cells and no TRPM2 protein in mitochondria from blank HEK293 cells (supplementary Fig.10c). Consistently, ADPR induced a robust increase in the Zn^2+^ level in mitochondria from hTRPM2-expressing, but not blank HEK293 cells (Fig. 6c,d). Collectively, these results are in support of mitochondrial expression of the TRPM2 channel and an important role in mitochondrial Zn^2+^ accumulation.

### PKC and NOX in Aβ_42_-induced hippocampal neurotoxicity and generation of ROS

**Fig. 7:**
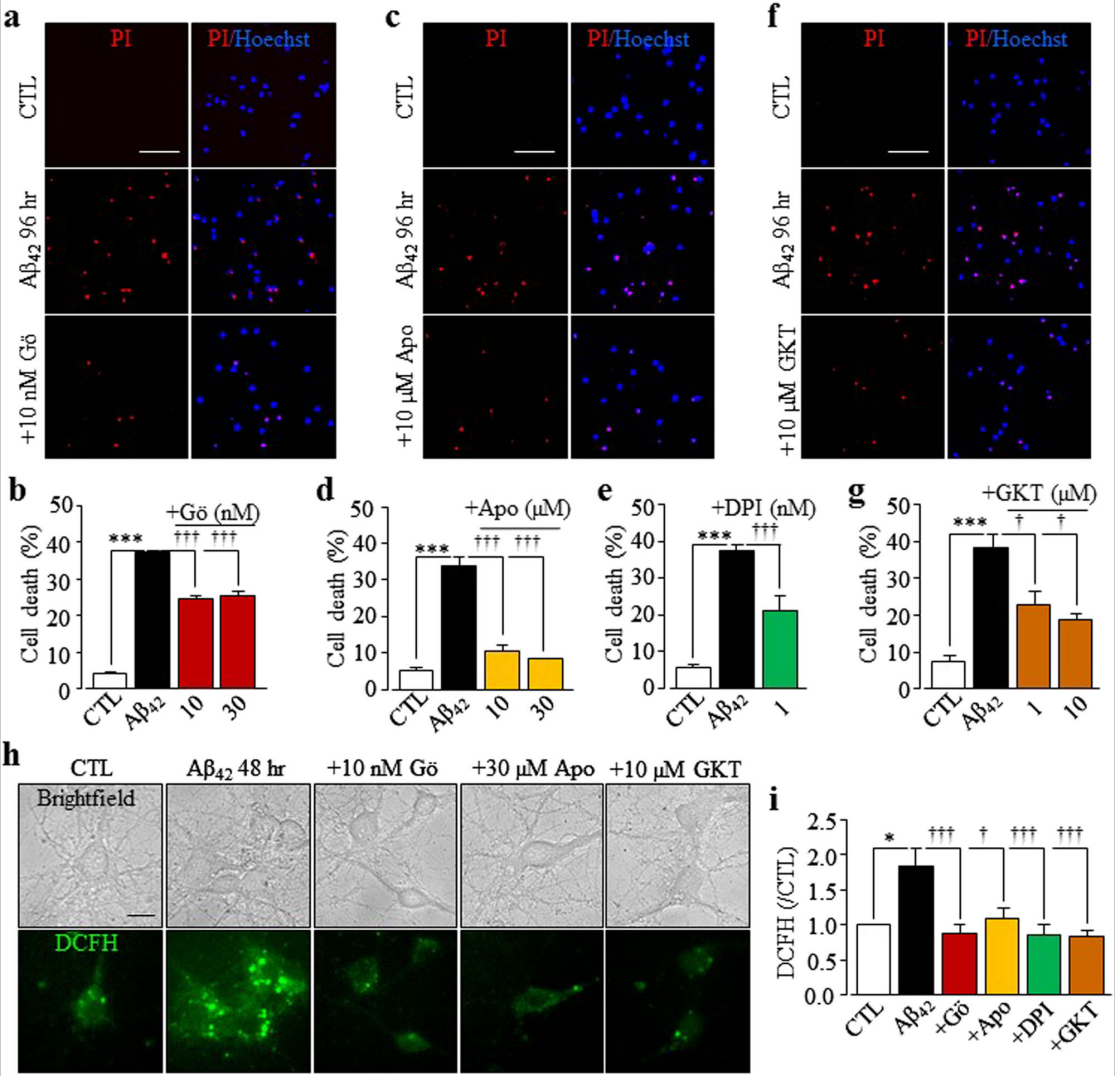
PKC and NOX inhibitors suppress Aβ_42_-induced neurotoxicity and generation of ROS in hippocampal neurons. **a**, **c**, **f** Representative images showing PI staining of wild-type neurons under control (CTL) conditions or after exposure to 1 µM Aβ_42_ for 48 h with or without treatment with 10 nM Gö6976 (Gö, **a**), 30 µM apocynin (Apo, **b**), or 10 µM GKT137831 (GKT, **c**), 30 min prior to and during exposure to Aβ_42_. Each consists of PI images showing dead neurons and merged images of Hoechst (blue)/PI staining showing all and dead neurons. Scale bar is 100 µm. **b**, **d**, **e**, **g** Summary of the mean percentage of PI-positive neurons under indicated conditions, from three to five independent experiment with each examining 400–600 neurons for each condition. ***p < 0.05 indicates difference from neurons under CTL conditions. ^†^*p* < 0.05 and ^†††^*p* < 0.005 indicate difference from neurons exposed to Aβ_42_ alone. **h** Representative images showing DCFH fluorescence in neurons under CTL and after exposure to 1 µM Aβ_42_ for 48 h with or without treatment with 10 nM Gö6976 (Gö), 30 µM apocynin (Apo), 1 nM DPI, or 10 µM GKT137831 (GKT), 30 min prior to and during exposure to Aβ_42_. Scale bar is 10 µm. **i** Summary of the mean DCFH fluorescence intensity in neurons under indicated conditions normalized to the basal or CTL level, from four independent experiments with each experiment examining 10–15 neurons for each conditions. *p < 0.05 indicates difference from CTL. ^†^*p* < 0.05; and ^†††^*p* < 0.005 indicate difference from that in neurons exposed with Aβ_42_ alone

**Fig. 8:**
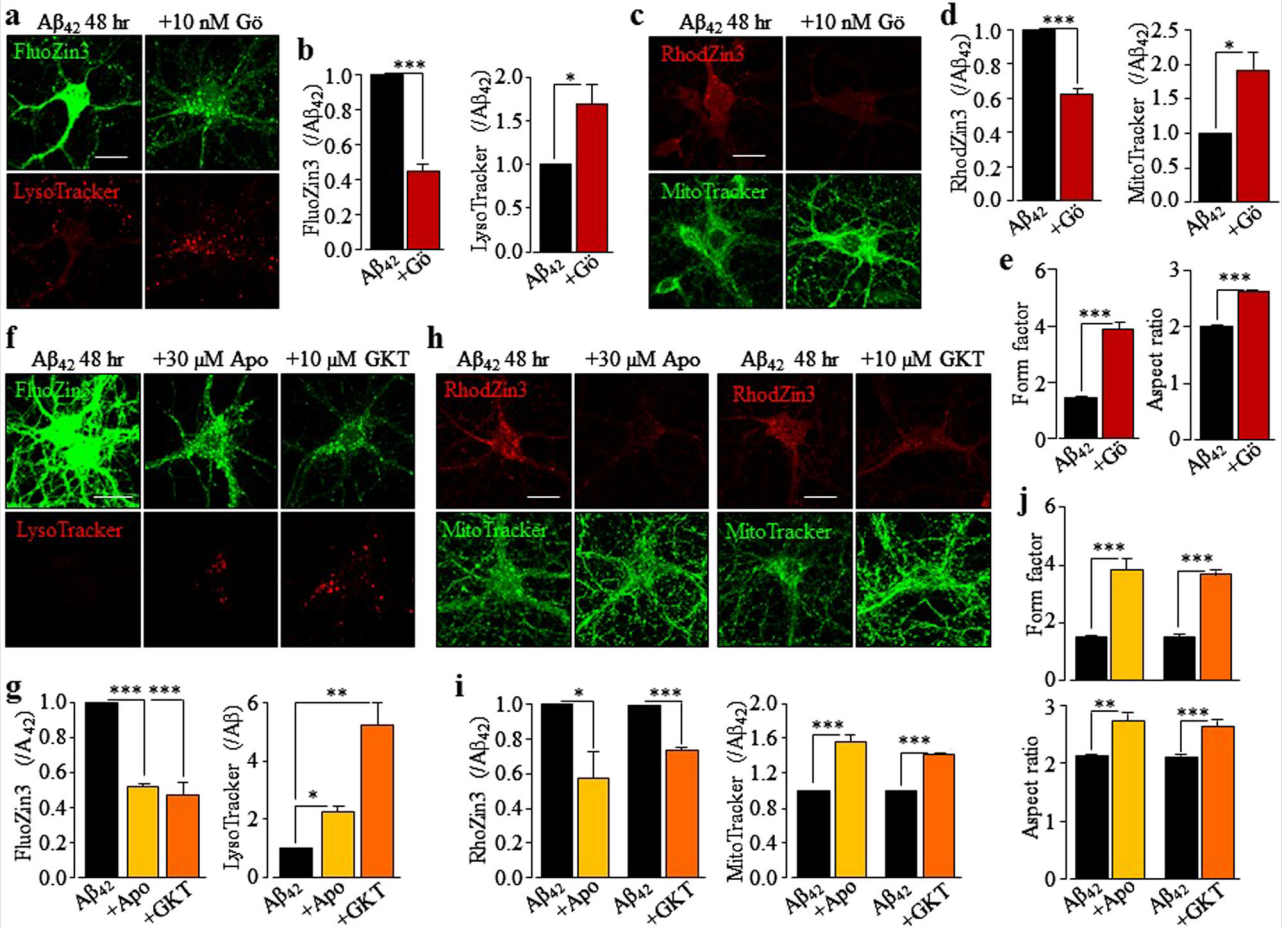
PKC and NOX inhibitors prevent Aβ_42_-induced increase in the [Zn^2+^]_c_, lysosomal dysfunction, mitochondrial Zn^2+^ accumulation, loss of mitochondrial function and change in mitochondrial morphology in hippocampal neurons. **a**, **f** Representative confocal images showing FluoZin3 (green) and LysoTracker (red) staining of wild-type neurons after exposure to 1 µM Aβ_42_ for 48 h with or without treatment with 10 nM Gö6976 (Gö, **a**), 30 µM apocynin (Apo) or 10 µM GKT137831 (GKT) (**f**), 30 min prior to and during exposure to Aβ_42_. Scale bar is 10 µm. **b**, **g** Summary of the mean fluorescence intensity of FluoZin3 or LysoTracker under indicated conditions normalized to that in neurons exposed to Aβ_42_ alone, from three to four independent experiments with each experiment examining 10–12 neurons for each condition. *p < 0.05; **p < 0.01; and ***p < 0.005 indicate difference from neurons exposed with Aβ_42_ alone. **c**,** h** Representative confocal images showing RhodZin3 (red) and MitoTracker (green) staining in hippocampal neurons exposed to 1 µM Aβ_42_ for 48 h with or without 10 nM Gö6976 **c**, 30 µM apocynin or 10 µM GKT137831 **h**. Scale bar is 10 µm. **d**, **i** Summary of the mean fluorescence intensity of RhodZin3 and MitoTracker under indicated conditions normalized to normalized to that in neurons exposed with Aβ_42_ alone. **e**, **j** Summary of the mean form factor and aspect ratio values of mitochondria under indicated conditions. The data were from three to four independent experiments with each experiment examining 15–20 neurons. *p < 0.05; **p < 0.01; and ***p < 0.005 indicate difference from neurons exposed with Aβ_42_ alone

NOX is an important source of ROS that induce neuronal cell death and protein kinase C (PKC) is known to activate NOX. Next, we examined whether PKC and NOX were engaged in Aβ_42_-induced neurotoxicity by determining the effects of Gö6976, a PKC inhibitor, apocynin and DPI, two generic NOX inhibitors, and GKT137831, a NOX1/4-selective inhibitor, on Aβ_42_-induced neurotoxicity and generation of ROS. Aβ_42_-induced neurotoxicity was significantly attenuated or prevented by treatment with 10–30 nM Gö6967, 10–30 µM apocynin, 1 nM DPI, or 1–10 µM GKT137831 (Fig. 7a-g). We also showed using DCFH-DA assay that Aβ_42_ induced a salient increase in cellular ROS, which was abolished by treatment with 10 nM Gö6976, 30 µM apocynin, 1 nM DPI, or 10 µM GKT137831 (Fig. 7h,i). To further investigate the role of PKC/NOX-mediated generation of ROS in Aβ_42_-induced neurotoxicity, we also examined the effects of inhibiting PKC and NOX on Aβ_42_-induced increase in the [Zn^2+^]_c_, lysosomal dysfunction, mitochondrial Zn^2+^ accumulation, and subsequent effects on mitochondrial function. Treatment with 10 nM Gö6976 strongly inhibited Aβ_42_-induced increase in the [Zn^2+^]_c_ (Fig. 8a,b), lysosomal dysfunction (Fig. 8a,b), mitochondrial Zn^2+^ accumulation (Fig. 8c,d), loss of MitoTracker intensity (Fig. 8c,d), and change in mitochondrial morphology (Fig. 8c,e; supplemental Fig.11a). Similarly, treatment with 30 µM apocynin or 10 µM GKT137831 resulted in a strong inhibition of Aβ_42_-induced increase in the [Zn^2+^]_c_ (Fig. 8f,g), lysosomal dysfunction (Fig. 8f,g), mitochondrial Zn^2+^ accumulation (Fig. 8h,i), loss of MitoTracker intensity (Fig. 8h,i), and change in mitochondrial morphology (Fig. 8h,j; supplementary Fig.11b-c). Overall, these results provide strong evidence to suggest that PKC and NOX play an important role in Aβ_42_-induced generation of ROS that lead to loss of lysosomal and mitochondrial functions and neurotoxicity.

### MEK/ERK in Aβ_42_-induced hippocampal neurotoxicity

**Fig. 9:**
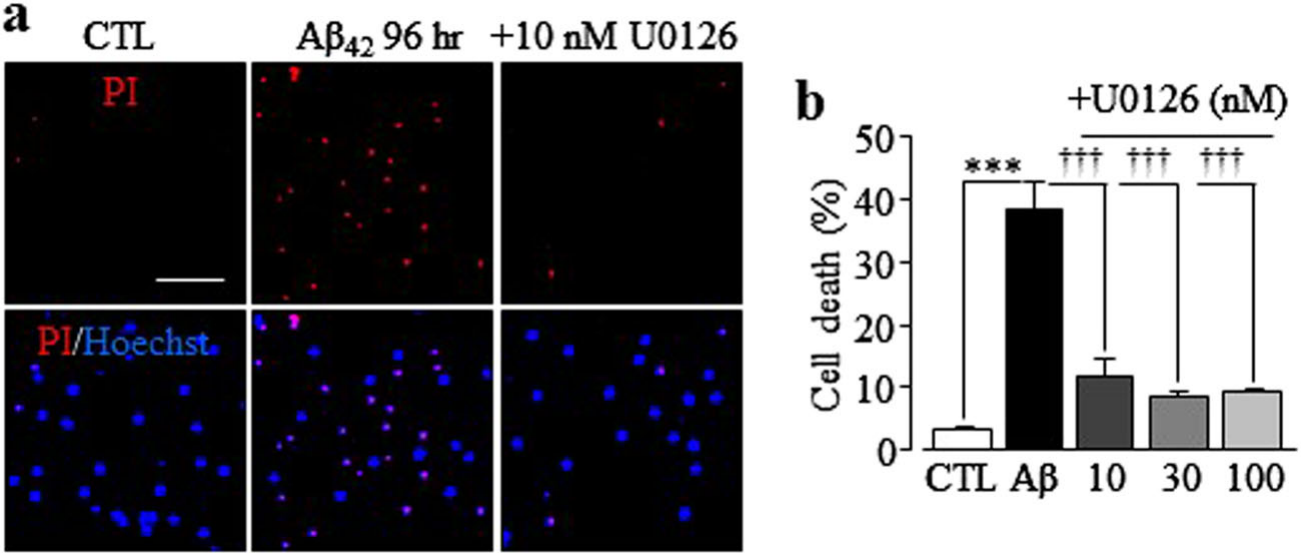
MEK/ERK inhibitor prevents Aβ_42_-induced hippocampal neurotoxicity. **a** Representative images showing PI staining of wild-type neurons under control (CTL) or after exposure to 1 µM Aβ_42_ for 96 h with or without treatment with 10 nM U0126, 30 min prior to and during exposure to Aβ_42_. Each panel consists of PI-staining image (red) showing dead neurons and merged Hoechst (blue)/PI staining showing all and dead neurons. Scale bar is 100 µm. **b** Summary of the mean percentage of PI-positive neurons under indicated conditions, from three independent experiments with each examining 400–650 neurons for each condition. ***p < 0.005 indicates difference from neurons under control conditions. ^†††^*p* < 0.005 indicates difference from neurons exposed with 1 µM Aβ_42_ alone

Activation of PARP-1 is long known as an important factor in ROS-induced neurotoxicity^40^. As introduced above, PARP-1-dependent generation of ADPR represents a major mechanism in ROS-induced TRPM2 channel activation and subsequent cell death^20^. A recent study has reported that ROS stimulates PARP-1 activation via mitogen-activated protein kinase (MEK) and downstream extracellular signal-regulated kinase (ERK) ^41^. Therefore, we finally examined the role of MEK/ERK in Aβ_42_-induced neurotoxicity. Aβ_42_-induced neurotoxicity was almost completely prevented by treatment with 10 nM U0126, a MEK/ERK inhibitor (Fig. 9a,b), thus suggesting critical engagement of MEK/ERK in Aβ_42_-induced neurotoxicity.

## Discussion

**Fig. 10:**
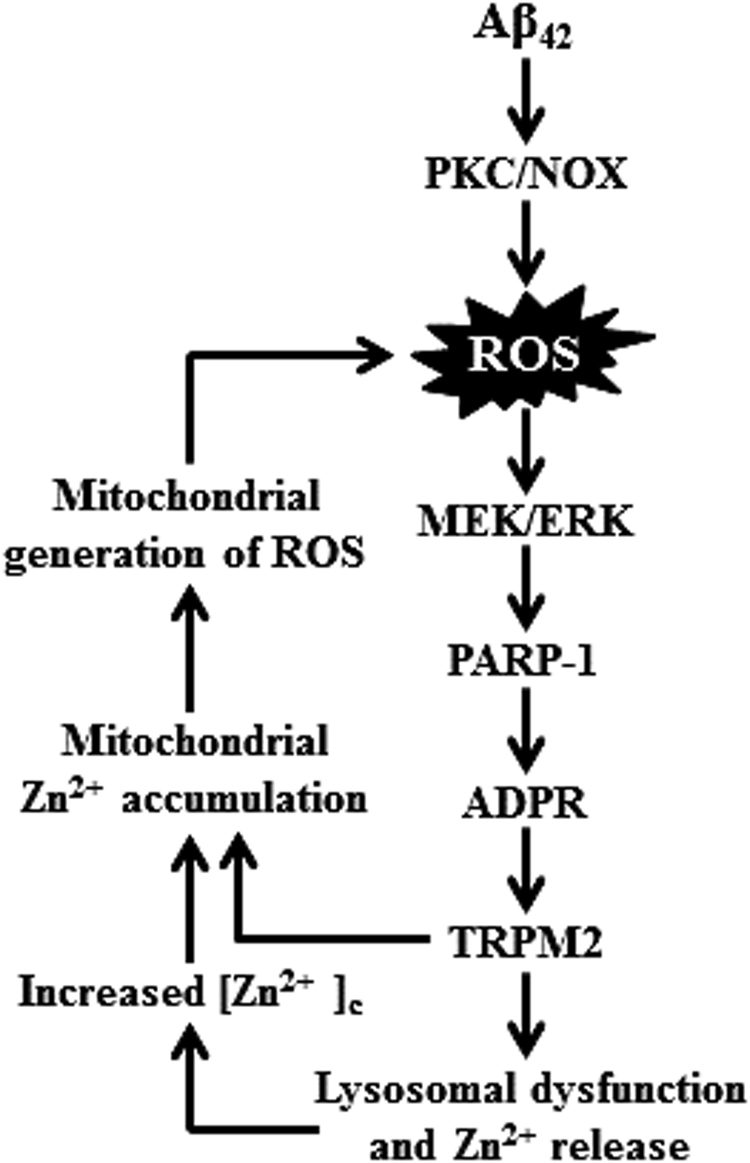
Schematic summary of the molecular mechanisms in Aβ_42_-induced activation of the TRPM2 channel and neurotoxicity in hippocampal neurons. Exposure to Aβ_42_ initiates generation of ROS via protein kinase C (PKC) and NADPH-dependent oxidases (NOX). ROS induces sequential activation of mitogen-activated protein kinases (MEK/ERK) and poly(ADP-ribose) polymerase-1 (PARP-1), generation of ADP-ribose (ADPR) and activation of the TRPM2 channel, and results in lysosomal dysfunction and Zn^2+^ release to increase the cytosolic Zn^2+^ concentration ([Zn^2+^]_c_). TRPM2-dependent mitochondrial Zn^2+^ accumulation causes loss of mitochondrial function and mitochondrial generation of ROS that further elevates the ROS level to form a positive feedback loop, which ultimately drives loss of lysosomal and mitochondrial functions and leads to neuronal death

The present study reveals multiple mechanisms that form a positive feedback loop to drive Aβ_42_-induced TRPM2-dependent hippocampal neurotoxicity (Fig. 10). Our findings provide novel and mechanistic insights into the causative relationship of TRPM2 channel with AD.

Consistently with Aβ-induced oxidative stress and TRPM2 as an oxidative stress-sensitive channel, recent studies describe distinctive cellular mechanisms by which Aβ-induced TRPM2 channel activation contributes to AD, including neurotoxicity^32^, neurovascular dysfunction^42^, and neuroinflammation^32,43^. Here we provide evidence to show a vital role for the TRPM2 channel in Aβ_42_-induced hippocampal neurotoxicity (Fig. 1). Ca^2+^ is known as an intracellular signal that is important for diverse cell functions, including cell death, and TRPM2 channel has been shown to play a role in ROS-induced Ca^2+^ signaling^15,44,45^. ROS-induced TRPM2-mediated cortical neuronal death was attenuated in extracellular Ca^2+^-free solution^25^. Although previous studies showed cell surface expression of the TRPM2 channel on hippocampal pyramidal neurons^30,46^, there was no measurable increase in the [Ca^2+^]_c_ following 24–48 h exposure to Aβ_42_ in our hippocampal neuronal preparations (supplementary Fig.12). Increasing evidence from recent studies examining hippocampal pyramidal neurons^30^, pancreatic β-cells^35^, and endothelial cells^36^ supports an important role of TRPM2-dependent increase in the [Zn^2+^]_c_ in ROS-induced cell death. Here we showed that Aβ_42_-induced hippocampal neurotoxicity was almost completely prevented by 100 nM TPEN acting as a selective Zn^2+^ chelator^34^ (Fig. 1d) as well as by TRPM2-KO or inhibition of the TRPM2 channel (Fig. 2a-d). Taken together, our results support that TRPM2-dependent increase in the [Zn^2+^]_c_ is critical in Aβ_42_-induced neurotoxicity.

Zn^2+^ is of particular abundance in hippocampal neurons and known to accumulate in degenerating neurons after ischemia or seizure^34,47^. Within the cytosol, Zn^2+^ is buffered by metallothioneins^12,14^ or stored in lysosomes and other intracellular organelles^13,35,48^. We found that Zn^2+^ in hippocampal neurons was mainly located in lysosome (supplementary Fig.4). In addition to a salient increase in the [Zn^2+^]_c_, Aβ_42_ elicited loss of lysosomal function evidenced by significant reduction in LysoTracker and AO intensity (Fig. 2a,b and supplementary Fig.5). This is consistent with that Aβ_42_ induces generation of ROS, as discussed below, and that ROS causes lysosomal dysfunction^13^ in hippocampal neurons. Taken together, these observations lead us to hypothesize that lysosomal dysfunction results in, at least in part, lysosomal Zn^2+^ release. This notion is supported by the observation that bafilomycin-induced lysosomal dysfunction also increased the [Zn^2+^]_c_ (supplementary Fig.9). In addition, bafilomycin-induced increase in the [Zn^2+^]_c_ was prevented by TRPM2-KO (supplementary Fig.8), indicating that lysosomal Zn^2+^ release is TRPM2-dependent. It has been proposed that lysosomal TRPM2-mediated Zn^2+^ release contributes to ROS-induced increase in the [Zn^2+^]_c_ and cell death in pancreatic β-cells^35^. However, it remains challenging to demonstrate whether or not TRPM2 is a Zn^2+^-permeable channel, due to the potent inhibition of TRPM2 channel by extracellular Zn^2+^ at high micromolar concentrations^49^. Regardless, further studies are required to better understand the mechanisms responsible for TRPM2-dependent lysosomal Zn^2+^ release in hippocampal neurons. Of notice, Aβ_42_-elicited lysosomal dysfunction in hippocampal neurons was prevented by TPEN (Fig. 2c-f) as well as TRPM2-KO or inhibition of the TRPM2 channel (Fig. 2a-d). These results suggest that TRPM2-dependent increase in the [Zn^2+^]_c_ has a reciprocal effect on lysosomal function.

The present study showed that Aβ_42_ induced considerable mitochondrial Zn^2+^ accumulation in hippocampal neurons (Fig. 3a,b). In remarkable resemblance with Aβ_42_, exposure to bafilomycin led to mitochondrial Zn^2+^ accumulation. Strikingly, the mitochondrial Zn^2+^ accumulation induced by both agents was prevented by TPEN (Fig. 3g-i) as well as by TRPM2-KO (Figs. 3a,b and 5a-d) or inhibition of the TRPM2 channel (Fig. 3d-f). Taken together, these results support the notion that lysosomal dysfunction and accompanying Zn^2+^ release give rise to mitochondrial Zn^2+^ accumulation. The finding that bafilomycin-induced mitochondrial Zn^2+^ accumulation was strongly dependent of the TRPM2 channel raised an intriguing question towards the TRPM2 channel in mitochondrial Zn^2+^ accumulation. Both immunostaining and western blotting suggest mitochondrial location of TRPM2 in hippocampal neurons (supplementary Fig.10). Consistently, ADPR stimulated Zn^2+^ accumulation in mitochondria isolated from WT but not TRPM2-KO hippocampal neurons (Fig. 6a,b). There was no ADPR-induced Zn^2+^ accumulation in isolated mitochondria in the absence of Ca^2+^ (Fig. 6b), consistent with Ca^2+^ being critical in ADPR-induced TRPM2 channel activation^50,51^, particularly in hippocampal neurons^30,46^. Similar findings were made regarding mitochondrial localization of TRPM2 (supplementary Fig.10c) and ADPR-induced Zn^2+^ accumulation in mitochondria isolated from HEK293 cells overexpressing the hTRPM2 channel, but not from blank HEK293 cells (Fig. 6c,d). Collectively, these results support the notion that the TRPM2 channel is located in mitochondria and plays an important role in mitochondrial Zn^2+^ accumulation. A precedent was made by a previous study showing involvement of the TRPC3 channel, another member of the TRP superfamily, in mitochondrial Ca^2+^ homeostasis^52^. However, as discussed above, it remains uncertain whether the TRPM2 channel permeates Zn^2+^. Further investigations are thus required to elucidate Aβ_42_-induced activation of the mitochondrial TRPM2 channel and associated mechanisms in mediating mitochondrial Zn^2+^ accumulation.

We also observed that Aβ_42_ induced strong loss of MitoTracker fluorescence (Fig. 3a,b) and measurable release of Cyt-c (supplementary Fig.6), suggesting Aβ_42_-induced loss of mitochondrial function. This is further supported by the finding that Aβ_42_-induced mitochondrial generation of ROS (Fig. 4a). Moreover, Aβ_42_ induced salient change in mitochondrial morphology (Fig. 3a-c), indicating altered mitochondrial dynamics and further studies are required to better understand the implication to mitochondrial function and Aβ_42_-induced neurotoxicity. Nonetheless, all these Aβ_42_-induced mitochondrial effects clearly depend on the TRPM2 channel, as they were prevented by TRPM2-KO or inhibition of the TRPM2 channel as well as by TPEN (Fig. 3d-i). These results strongly support the causative relationship of rising [Zn^2+^]_c_ with loss of mitochondrial function and generation of ROS reported by earlier studies^14,53^. Furthermore, these results provide clear evidence for the first time to show a critical role for the TRPM2 channel or TRPM2-dependent mitochondrial Zn^2+^ accumulation in Aβ_42_-induced loss of mitochondrial function and mitochondrial generation of ROS. We propose that mitochondrial generation of ROS acts as a positive feedback in Aβ_42_-induced hippocampal neurotoxicity (Fig. 10).

It is well-known that NOX-mediated generation of ROS plays a crucial role in neurotoxicity implicated in ischemic stroke and AD^10,11,54^. As shown here, Aβ_42_-induced neurotoxicity (Fig. 7c-g) and cellular oxidative stress (Fig. 7h,i) were strongly suppressed or abolished by apocynin, DPI, or GKT137831, supporting a role of NOX, particularly NOX1 and/or NOX4, in Aβ_42_-induced generation of ROS, as reported in recent studies in hippocampal neuronal death following stroke^54–56^. Notably, Aβ_42_-induced neurotoxicity (Fig. 7a,b) and oxidative stress (Fig. 7h,i) were inhibited by Gö6976, suggesting a role for PKC in Aβ_42_-induced activation of NOX. Furthermore, Aβ_42_-induced lysosomal dysfunction and increase in the [Zn^2+^]_c_, mitochondrial Zn^2+^ accumulation, loss of mitochondrial function, and change in mitochondrial morphology (Fig. 8; supplementary Fig.11) were largely reversed by inhibition of NOX. These results indicate that activation of NOX is an important mechanism that likely initiates Aβ_42_-induced generation of ROS, which subsequently induces activation of the PARP-1 and TRPM2 channel, lysosomal dysfunction and Zn^2+^ release, an increase in the [Zn^2+^]_c_, and mitochondrial Zn^2+^ accumulation (Fig. 10).

It is well-documented that PARP-1 is critical in ROS-induced TRPM2 channel activation and cell death in various cell types, including Aβ_42_-induced neuronal death^24^. As recently shown, ROS stimulates PARP-1 via MEK/ERK^41^, and ERK is engaged in PARP-1 activation and oligodendrocyte death induced by transient ischemia^57^. Here, we showed that Aβ_42_-induced neurotoxicity in hippocampal neurons was completely prevented by U0126 (Fig. 9a,b), indicating that MEK/ERK is critical in Aβ_42_-induced PARP-1 activation in hippocampal neurons (Fig. 10).

In summary, this study reveals multiple mechanisms, including PKC/NOX-mediated generation of ROS, activation of MEK/ERK and PARP-1, lysosomal dysfunction and Zn^2+^ release, mitochondrial Zn^2+^ accumulation, loss of mitochondrial function, and mitochondrial generation of ROS, that are critically engaged in forming a positive feedback loop that drives Aβ_42_-induced TRPM2 channel activation and loss of lysosomal and mitochondrial function, which ultimately leads to hippocampal neurotoxicity. These findings provide novel and mechanistic insights into AD pathogenesis.

## Materials and Methods

### Reagents

All reagents, including 2-APB (2-aminoethoxydiphenyl borate), DPQ (3,4-dihydro-5[4-(1-piperindinyl)butoxy]-1(2 H)-isoquinoline), TPEN (N,N,N’,N’-tetrakis(2-pyridylmethyl)ethylenediamine), DPI (diphenyleneiodonium), and DCFH-DA (2′,7′-dichloro-dihydro-fluorescein diacetate), were commercially purchased from Sigma unless specifically indicated. All stock solutions including the Aβ_42_ and Aβ_42-1_ peptides were prepared following the manufacturers’ instructions, aliquoted and kept at −20 °C.

### Primary hippocampal neuron culture preparation

All experiments and experimental protocols involving mice were approved by the University of Leeds Ethical Review Committee and performed in accordance with the University of Leeds guidelines and procedure and conforming to the UK Home Office rules and regulations. Generation of transgenic TRPM2-KO C57BL/6 mice was detailed previously^58^. Primary hippocampal neurons were prepared from early postnatal (P0–P1) WT C57BL/6 mice and TRPM2-KO mice using the protocols described previously^59^. In brief, hippocampal tissues were dissected from the whole brain and collected into a 3.5-cm petri-dish containing ice-cold Hank’s balanced salt solutions (HBSS, Invitrogen). Tissues were incubated in 2 ml of 0.125% trypsin-ethylenediaminetetraacetic acid (trypsin-EDTA) solutions (Invitrogen) in 37°C for 15 min, stirred up by gentle swirling every 5 min. After trypsin-EDTA solutions were removed, the tissues were transferred in 2 ml of Dulbecco’s Modified Eagle Medium: Nutrient Mixture F-12 (DMEM/F12) containing 10% horse serum (Thermo Scientific) and carefully triturated by pipetting 50 times. The dissociated tissues were filtered into a 50-ml Falcon tube through a 70-µm nylon cell strainer (Fisher Scientific) to obtain single-cell suspension. Cell suspension was centrifuged at 100 ×* g* for 5 min, and cell pellets were re-suspended in fresh DMEM/F12 medium supplemented with 10% horse serum (Thermo Scientific), 5 unit/ml penicillin and 50 µg/ml streptomycin. Single cells were seeded in poly-l-lysine pre-coated 24-well plate or glass-bottomed petri-dish at a density of 100 cells/mm^2^ in DMEM/F12 medium supplemented with 10% horse serum (Thermo Scientific), which was replaced after 4 h with Neurobasal® medium supplemented with 2% serum free B27^®^ supplement, 0.5 mM l-glutamine, 5 unit/ml penicillin and 50 µg/ml streptomycin. Cytosine β-d-arabinofuranoside was added at the final concentration of 1 µM after 2 days to inhibit microglial growth. Cells were cultured 14–16 days in vitro at 37°C under 5% CO_2_ humidified conditions, with the medium changed twice a week. Immunostaining with an antibody recognizing microtubule associated protein-2 (MAP-2), a neuron-specific protein, showed 98% cells in hippocampal neuronal preparations used in this study were MAP-2 positive.

### Immunocytochemistry

Neurons were seeded in poly-l-lysine coated coverslips inserted in 24-well plate. After gently rinsed with phosphate buffer saline (PBS), neurons were incubated for 1 h in Zamboni’s fixative solutions, made of 15% (v/v) picric acid and 5.5% (v/v) formaldehyde in PBS. Fixed cells were rinsed with PBS and incubated for 1 h with blocking solutions, made of 10% (v/v) goat serum and 4% (v/v) Triton X-100 in PBS. In some experiments, cells were incubated in 50 nM MitoTracker Red CMXRos (Life Technologies) for 30 min before fixing. Cells were incubated with primary rabbit anti-TRPM2 antibody (1:1000; Bethyl) or mouse anti-Cyt-c antibody (1:100, BD Pharmingen) overnight at 4 °C. Cells were washed in PBS, and incubated with anti-rabbit or anti-mouse IgG secondary antibody conjugated with fluorescein isothiocyanate for 1 h. Cells were washed with PBS and rinsed in water before mounted with the SlowFade Gold Antifade reagent (Invitrogen) and kept in 4°C. Images were captured using an inverted LSM880 confocal microscope with a ×63 objective (Zeiss). ImageJ software (National Institutes of Health, USA) was used for image analysis of fluorescent intensity.

### PI-staining assays

Neuronal death was examined using propidium iodide (PI) staining. In brief, following exposure to Aβ_42_ or Aβ_42-1_ (ChinaPeptides, Shanghai, China) under indicated conditions, neurons in culture medium were further incubated for 30 min that contained 5 µg/ml PI and 1 µM Hoechst 33342 (Cell Signaling Technology). In some experiments, inhibitors were added for 30 min before and during exposure to Aβ_42_. Images were captured using an EVOS Cell Imaging System (Life Technologies). ImageJ software was used for analysis of neurons stained with PI and Hoechst.

### Single-cell confocal imaging

Neurons seeded in glass-bottomed petri-dish (World Precision Instruments). After the culture medium was removed, neurons were rinsed with standard buffer solution (SBS: 130 mM NaCl, 1.5 mM CaCl_2_, 5 mM KCl, 1.2 mM MgCl_2_, 8 mM glucose, 10 mM HEPES, pH 7.4) and incubated in SBS containing 1 µM Fluo4-AM, 1 μM FluoZin3-AM or 3 µM RhodZin3-AM (Life Technologies) and 0.1% (w/v) pluronic acid at 37°C in a tissue culture incubator for 1 h. In some experiments, neurons were kept in SBS containing 25 nM MitoTracker Red CMXRos, 100 nM MitoTracker Green FM, or 1 µM LysoTracker Red DND-99 (all from Life Technologies) after removal of FluoZin3 or RhodZin3. Neurons were rinsed with and kept in SBS. Inhibitors were added into SBS at indicated concentrations to test their effects on the cytosolic or mitochondrial Zn^2+^ as well as lysosomal and mitochondrial functions. For time-lapse recording, images were captured every 5 min for a total duration of 30 min after bafilomycin was administrated. Neurons were maintained with SBS before images were captured using an inverted LSM880 confocal microscope with a ×63 objective (Zeiss). Environmental control was applied to maintain 37°C and 5% CO_2_ during live cell imaging. ImageJ software was used for analysis of fluorescent intensity.

### ROS generation

Mitochondrial ROS generation was measured using MitoTracker Red CM-H_2_Xros (Life Technologies) according to the manufacturer’s instructions. Cellular oxidative stress was monitored by DCFH-DA. After exposed to indicated treatments, neurons were incubated in culture medium containing 100 nM MitoTracker Red CM-H_2_Xros or 3 µM DCFH-DA for 30 min at 37 °C. Cells were washed with and maintained in SBS before images were captured using an EVOS Cell Imaging System. ImageJ software was used for analysis of fluorescent intensity.

### Mitochondria isolation and Zn^2+^ labeling

Mitochondria were isolated from cultured hippocampal neurons, human embryonic kidney 293 (HEK293) cells, TRPM2-inducible HEK293 cells overexpressing the recombinant human TRPM2 channel (hTRPM2-HEK293 cells)^60^, using a Mitochondria Isolation kit (Thermo Scientific) according to the manufacturer’s instructions. Isolated mitochondria were suspended with Mitochondria Isolation Reagent C from the kit and exposed to the  indicated treatments and, after centrifugation at 12,000 × *g* for 5 min, re-suspended in SBS containing 1 µM RhodZin3-AM^61^ with 0.1% pluronic acid (Life Technologies) and incubated at 37°C for 1 h. In some experiments, Ca^2+^-free SBS supplemented with 0.4 mM EGTA was used. RhodZin3-AM was removed by centrifugation at 12,000 ×* g* for 5 min, and pellets were re-suspended with SBS. The mitochondria suspension was dropped on a glass slide and covered with a rectangular coverslip. Images were captured using an inverted LSM700 confocal microscope with a ×63 oil objective (Zeiss). ImageJ software was used for analysis of fluorescent intensity.

### Analysis of lysosomal dysfunction

Lysosomal dysfunction or lysosomal membrane permeabilization was evaluated by AO staining. After treated under indicated conditions, neurons were stained with 5 μg/ml AO at 37 °C for 15 min. AO-induced red fluorescence were captured using an EVOS Cell Imaging System. ImageJ software was used for analysis of AO red fluorescence intensity.

Western blotting. Mitochondria were isolated from mouse hippocampus and cortex, blank HEK293 cells, hTRPM2-expressing HEK293 cells, as described above. Isolated mitochondria were lysed at 4°C in radioimmunoprecipitation assay buffer for 30 min. Proteins were separated by sodium dodecyl sulfate-polyacrylamide gel electrophoresis on 10% separating gels and transferred onto polyvinylidene difluoride membranes. After incubation with the primary rabbit anti-TRPM2 antibody (1:500; Abcam), mouse anti-LAMP-1 antibody (1:1000; Genetex) or mouse anti-Cyt-c antibody (1:500; BD Pharmingen), and the secondary anti-rabbit or anti-mouse antibodies conjugated to horseradish peroxidase. Proteins were visualized using SuperSignal West Pico PLUS Chemiluminescent Substrates (ThermoFisher).

### Data analysis

Neuronal death was expressed by the number of PI-positive neurons as percentage of all neurons in the same areas identified by Hoechst staining. Co-localization of two fluorescent signals was quantified by Pearson’s correlation coefficient that varies between 0 and 1, being no and total positive correlation, as described previously^62^. The morphology of mitochondria was characterized by computer-assisted analysis of the aspect ratio and form factor values as described previously^63^. Data are presented as mean ± standard error mean (S.E.M.). Statistical significance analysis was conducted using analysis of variance with post hoc Tukey test, with significance at the level of *p* < 0.05.

## Electronic supplementary material

supplementary data
