# Supplementary material for: Multiple molecular mechanisms form a positive feedback loop driving amyloid β42 peptide-induced neurotoxicity via activation of the TRPM2 channel in hippocampal neurons
Source: Cell Death Dis. 2018 Feb 7;9(2):195. doi: 10.1038/s41419-018-0270-1 (PMC5833848; doi:10.1038/s41419-018-0270-1)

Supplementary data for the manuscript by Li and Jiang

Fig. 1 Aβ$_{42}$ induces TRPM2-dependent hippocampal neuronal death. (**a**) Representative images showing PI staining of wild-type (WT) and TRPM2-KO neurons under control (CTL) conditions or after exposure to 100 ng/ml μM Aβ$_{42}$ for 24, 48 and 96 hr. Each panel consists of brightfield image showing neurons, PI staining image (red) showing dead neurons, and merged Hoechst (blue)/PI staining image showing all and dead neurons. Scale bar is 100 μm. **(b, c)** Summary of the mean percentage of PI positive neurons under indicated conditions from 3 independent experiments, with each experiment examining 300-450 neurons. Black and grey bars represent the percentage of neuronal death in WT and TRPM2-KO neurons, respectively. ***, $p < 0.005$ indicates significant difference from respective CTL. †††, $p < 0.005$ indicates significant difference between WT and TRPM2-KO neurons.

Fig. 2 Aβ$_{42}$ but not Aβ$_{42\text{-}1}$ induces hippocampal neuronal death. Representative images showing PI staining of wild-type (WT) neurons under control (CTL) conditions or after exposure to 1 μM Aβ$_{42}$ or Aβ$_{42\text{-}1}$ for 96 hr. Each panel consists of brightfield image showing neurons, PI staining image (red) showing dead neurons, and merged Hoechst (blue)/PI staining image showing all and dead neurons. Scale bar is 100 μm. The mean data is shown in Fig. 1c.

Fig. 3 PARP-1 inhibitors attenuates Aβ$_{42}$-induced hippocampal neuronal death. (a) Representative images showing PI staining of wild-type hippocampal neurons treated with 1 μM PJ34 or 30 μM DPQ, 30 min prior to and during exposure to 1 μM Aβ$_{42}$ for 96 hr. Each panel consists of brightfield image showing neurons, PI staining images showing dead neurons, and merged images showing Hoechst/PI staining for all and dead neurons. Scale bar is 100 μm. **(b)** Summary of the mean percentage of PI positive neurons from 3-4 independent experiments, with each experiment examining 350-450 neurons. ***, $p < 0.005$ indicates significant difference from control (CTL) or untreated neurons. †, $p < 0.05$ indicates difference from neurons exposed with Aβ$_{42}$ alone.

Fig. 4 Lysosomal localization of Zn^{2+} in hippocampal neurons. Representative confocal images showing co-staining of FluoZin3 with LysoTracker (top) or with MitoTracker (bottom) in wild-type hippocampal neurons. Scale bar is 10 μm. Similar results were observed in 3 or more independent experiments each examining 10-15 neurons.

Fig. 5 Aβ_{42} induces TRPM2-dependent lysosomal dysfunction in hippocampal neurons. **(a)** Representative images showing acridine orange (AO) staining of wild-type (WT) hippocampal neurons under control (CTL) conditions and after exposure to 100 nM bafilomycin for 15 min, or 1 µM Aβ_{42} for 48 and 96 hr. **(b)** Representative images AO staining of TRPM2-KO hippocampal neurons under CTL and after exposure to 1 µM Aβ_{42} for 48 and 96 hr. **(c)** Summary of the mean percentage of AO intensity under indicated conditions from 3 independent experiments, with each experiment examining 60-80 neurons. *, $p < 0.05$ indicates significant difference from CTL. Scale bar is 10 µm (a, b).

Fig. 6 Aβ_{42} induces cytochrome-C release in hippocampal neurons. **(a)** Representative images showing cytochrome-c (Cyt-c) and DAPI staining of wild-type (WT) hippocampal neurons under control (CTL) conditions and after exposure to 1 µM Aβ_{42} for 24 and 48 hr. Scale bar is 10 µm. **(b)** Summary of the mean percentage of Cyt-c fluorescence intensity under indicated conditions from 2 independent experiments, with each experiment examining 30-45 neurons. **, $p < 0.01$; and ***, $p < 0.001$ indicate significant difference from CTL.

Fig. 7 Aβ_{42} induces TRPM2-dependent loss of mitochondrial function, and change in the mitochondrial morphology. Representative confocal images from Fig. 3a are presented in large size to show MitoTracker Green staining of wild-type (WT) and TRPM2-KO hippocampal neurons under control (CTL) conditions and after exposure to 1 µM Aβ_{42} for 24 and 48 hr.

Fig. 8 Aβ_{42} induces TRPM2-dependent change in mitochondrial morphology in hippocampal neurons. Computer-assisted analysis of the aspect ratio and form factor of mitochondria in wild-type (WT) (a) or TRPM2-KO hippocampal neurons (b) under control (CTL) conditions (black close rectangle) exposed to 1 µM Aβ_{42} for 24 hr (red close circle) or 48 hr (blue close triangle), or in WT hippocampal neurons after exposure to 1 µM Aβ_{42} for 48 hr (red close circle) without (black close rectangle) or with treatment with 1 µM PJ34 (c), 10 µM 2-APB (d) or 100 nM TPEN (e), prior to and during exposure to Aβ_{42}.

Fig. 9 Bafilomycin induces TRPM2-dependent increase in the $[Zn^{2+}]_c$ in hippocampal neurons. **(a)** Representative images showing FluoZin3 staining of wild-type (WT) and TRPM2-KO hippocampal neurons under control (CTL) conditions and after exposure to 100 nM bafilomycin (Baf) for 30 min. Scale bar is 10 µm. **(b)** Summary of the mean percentage

of FluoZin3 intensity under indicated conditions from 40-60 neurons. ***, $p < 0.001$ indicate significant difference from CTL. NS, no significant difference.

Fig. 10 Expression of TRPM2 protein in mitochondria. (**a**) Representative confocal images showing TRPM2 immunostaining (green) and MitoTracker (red) in wild-type hippocampal neurons. Scale bar is 10 μm. (**b**) Summary of the mean Pearson's correlation coefficient from 3 independent experiments, with each experiment examining 10-15 neurons. (**c**) Representative western blot showing the TRPM2 expression in mitochondria isolated from blank HEK293 (HEK293), HEK293 overexpressing human TRPM2 (hTRPM2-HEK293), and mouse hippocampal and cortex. Note that mitochondrial preparations showed positive expression of cytochrome-c (Cyt-c) but the negative expression of lysosome-associated membrane protein 1 (LAMP-1) protein. Similar observations were observed in three independent experiments.

Fig. 11 PKC and NOX inhibitors prevent on Aβ$_{42}$-induced change in mitochondrial morphology in hippocampal neurons. Computer-assisted analysis of the aspect ratio and form factor of mitochondria in wild-type hippocampal neurons after exposed to 1 μM Aβ$_{42}$ for 48 hr without (black close circle) or with treatment with 10 nM Gö6976 (Go) (a, grey open rectangle), 10 μM apocynin (Apo) (b, yellow rectangle) or 10 μM GKT137831 (GKT) (c, green rectangle), 30 min prior to and during exposure to Aβ$_{42}$.

Fig. 12 Aβ$_{42}$ induces no change in the $[Ca^{2+}]_c$ in hippocampal neurons. (a) Representative confocal images showing Fluo4 staining (green) of wild-type hippocampal neurons under control (CTL) conditions or after exposure to 1 μM Aβ$_{42}$ for 24 or 48 hr, or 5 μM ionomycin for 5 min. Scale bar is 10 μm. (b) Summary of the mean Fluo4 intensity under indicated conditions. NS, no significant difference.

Supplementary Fig. 1

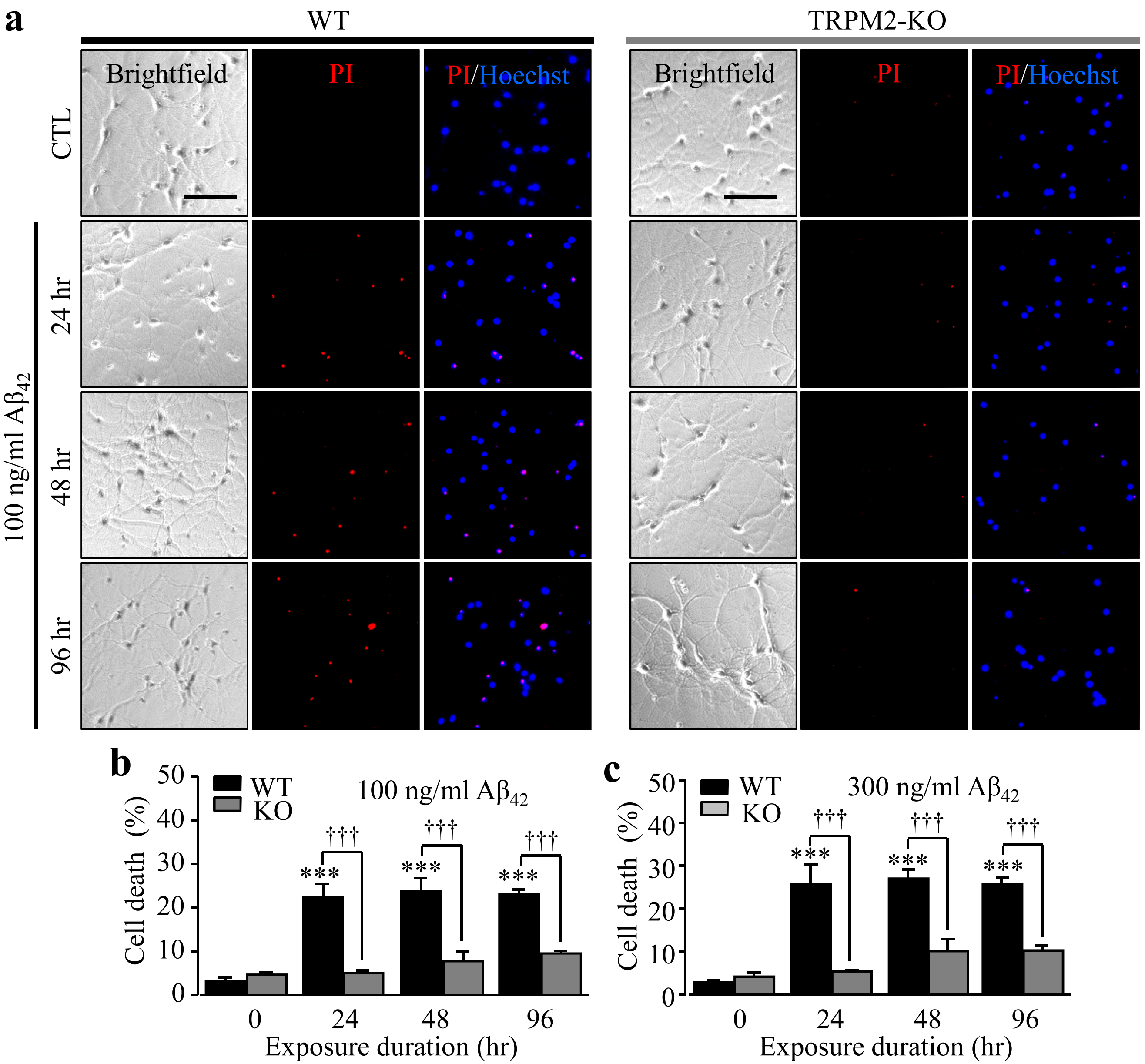

Supplementary Fig. 2

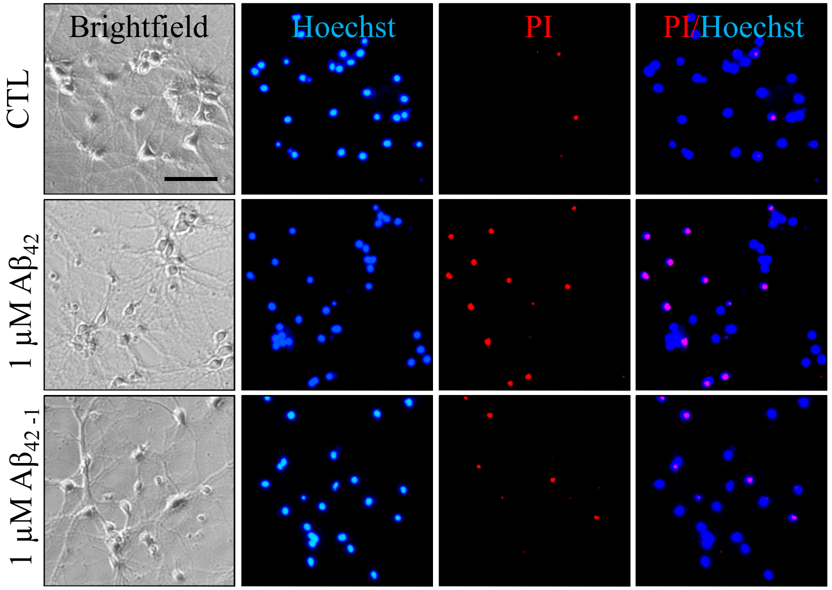

Supplementary Fig. 3

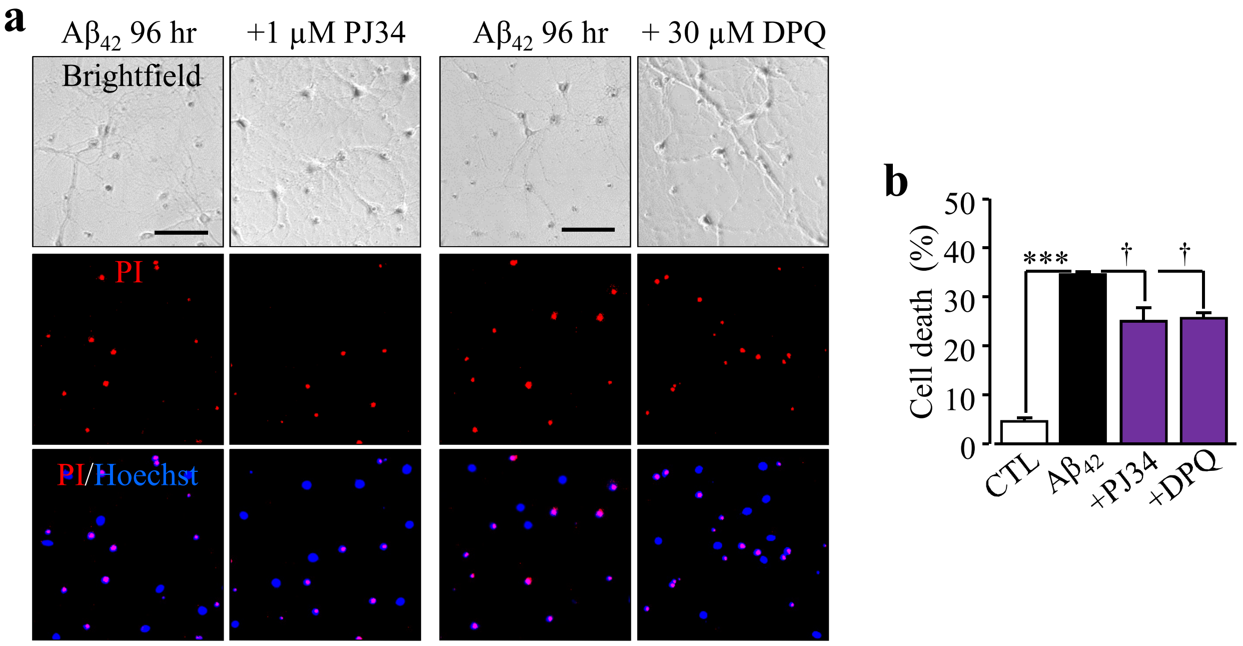

Supplementary Fig. 4

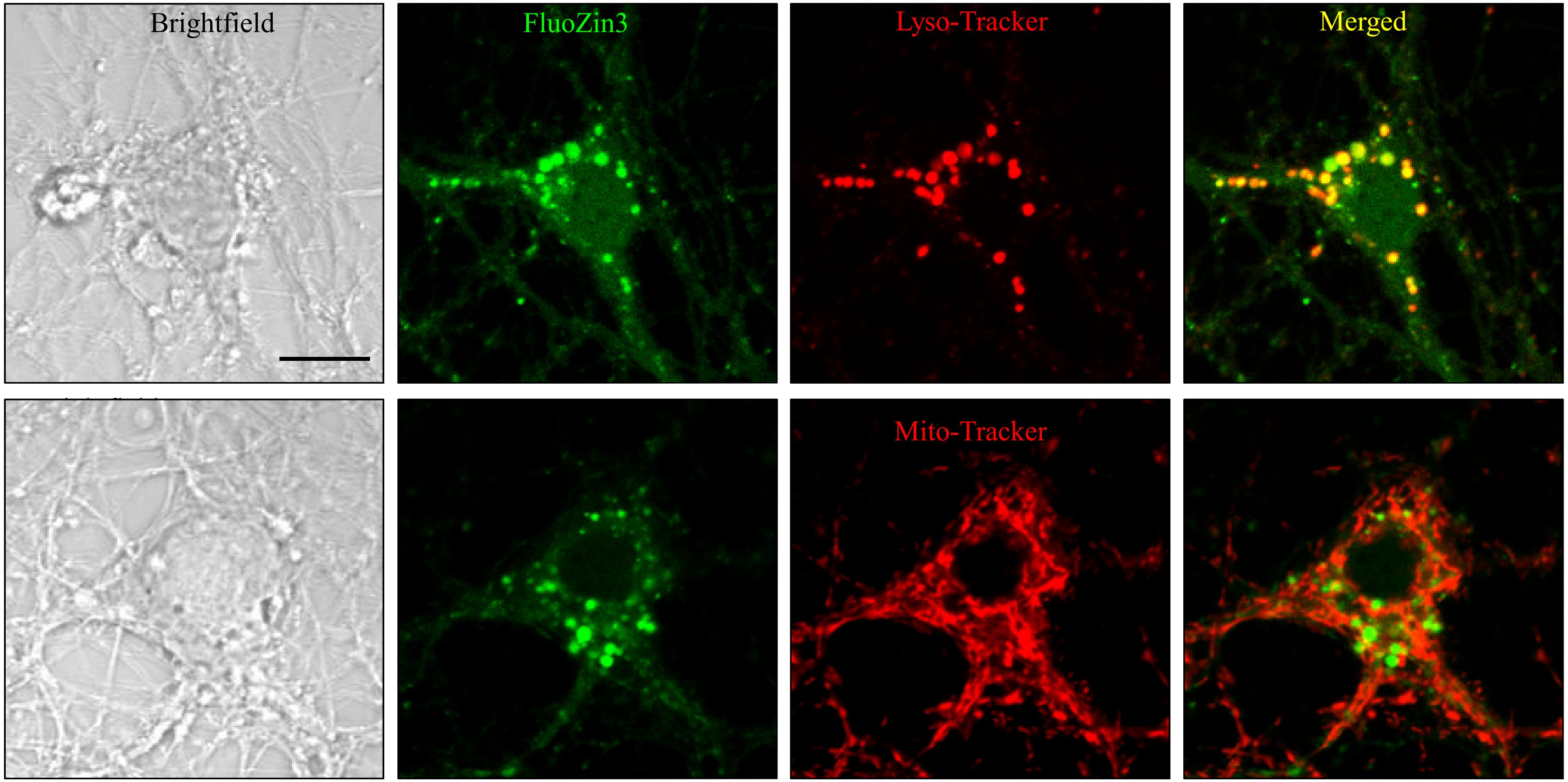

Supplementary Fig. 5

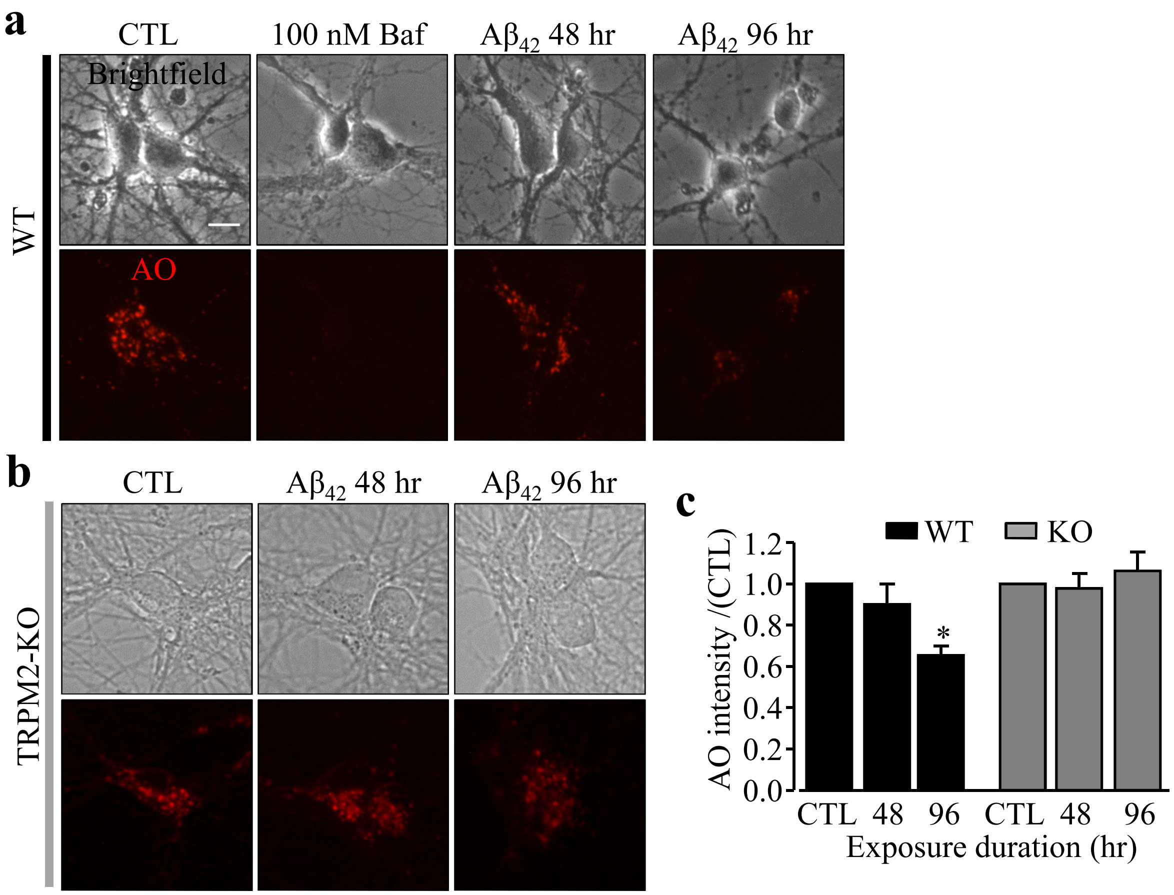

Supplementary Fig. 6

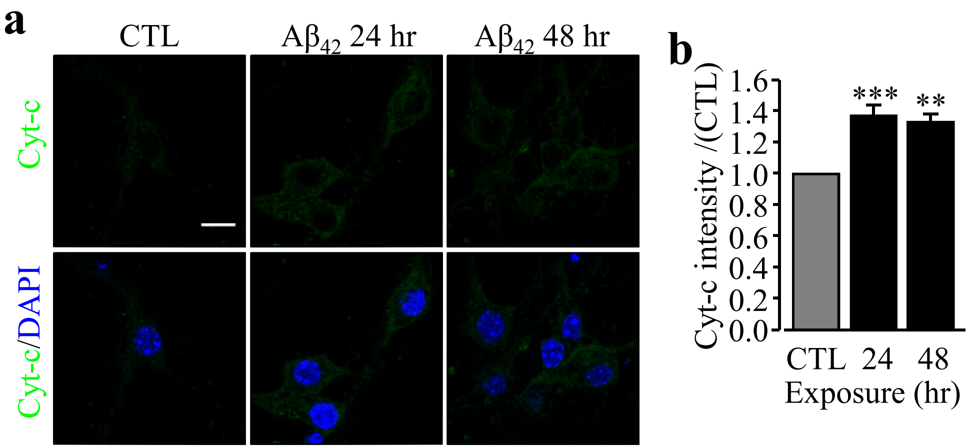

Supplementary Fig. 7

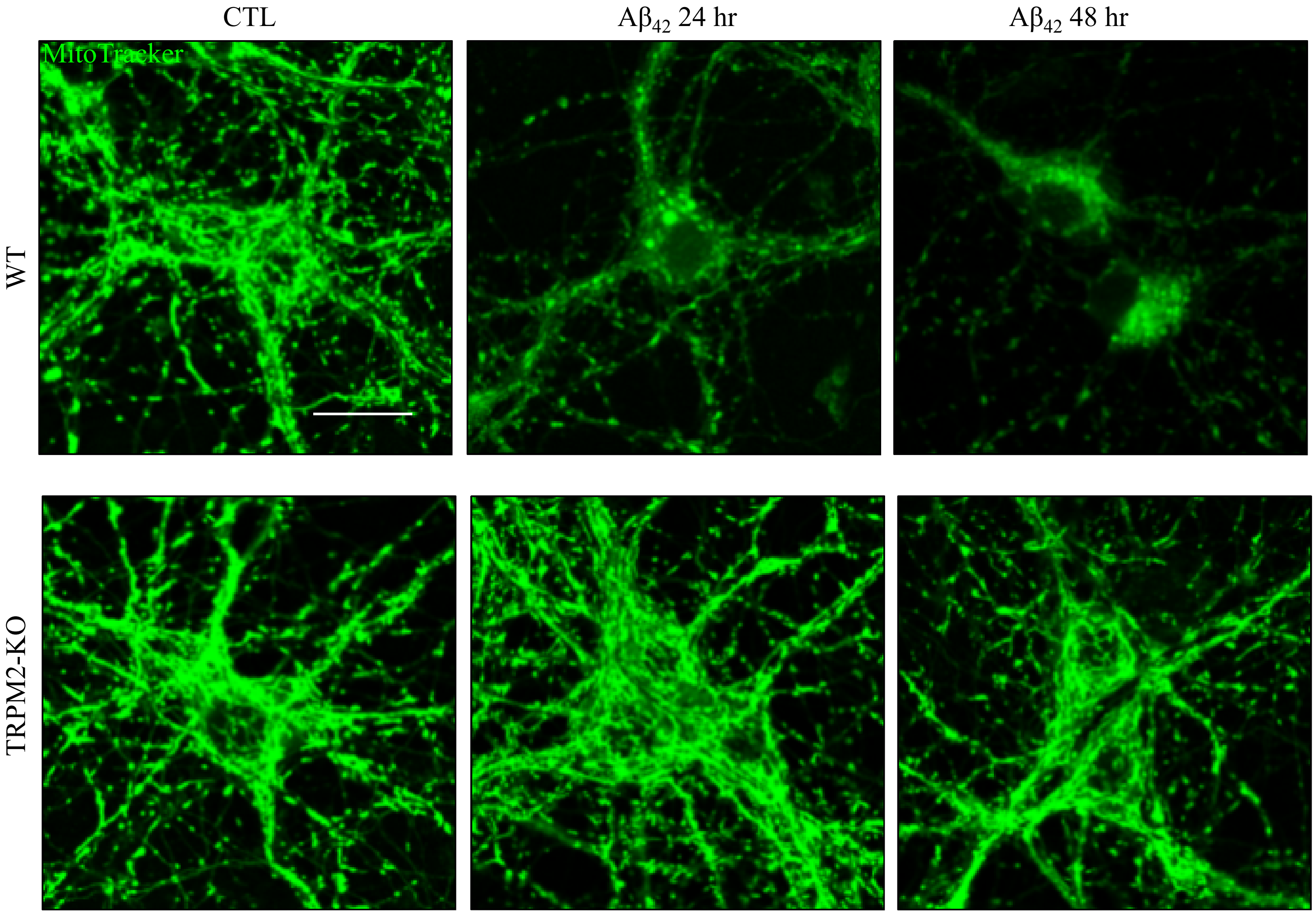

Supplementary Fig. 8

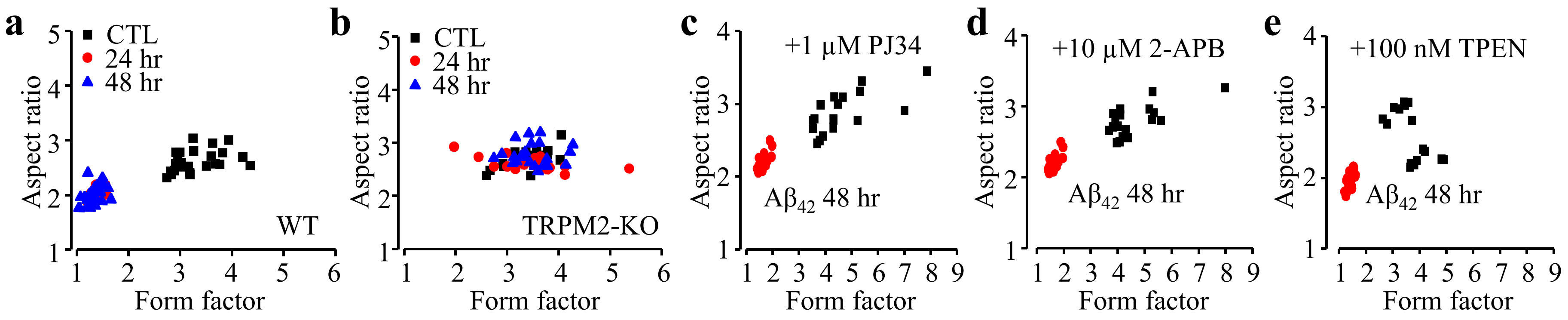

Supplementary Fig. 9

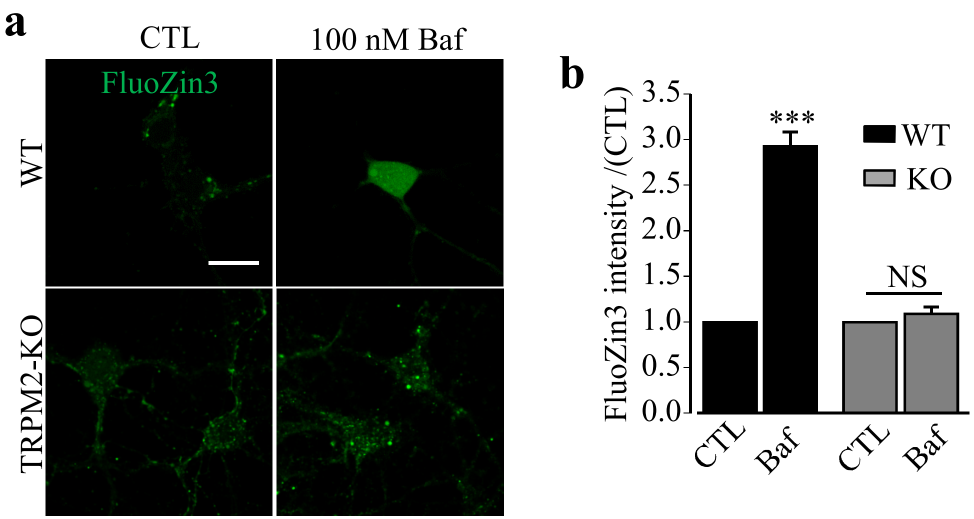

Supplementary Fig. 10

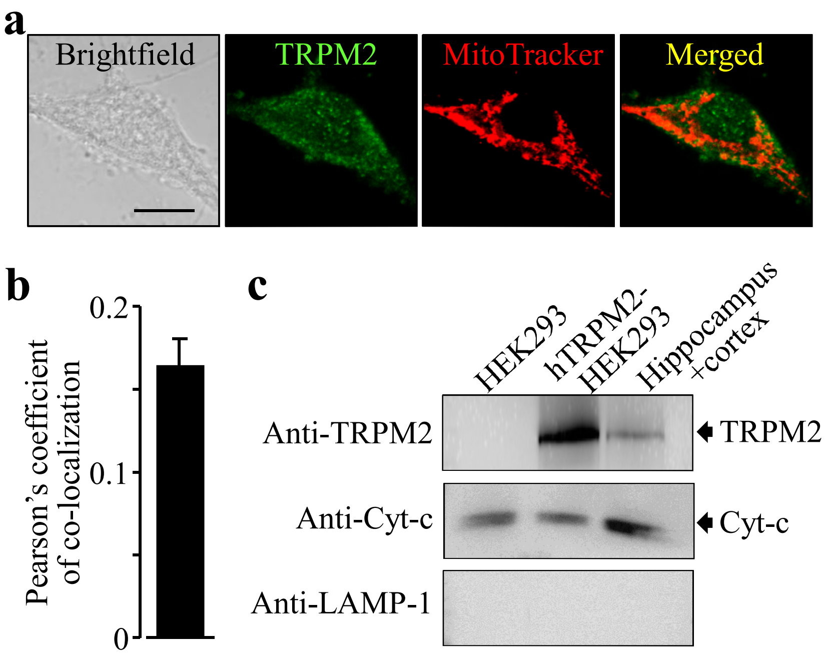

Supplementary Fig. 11

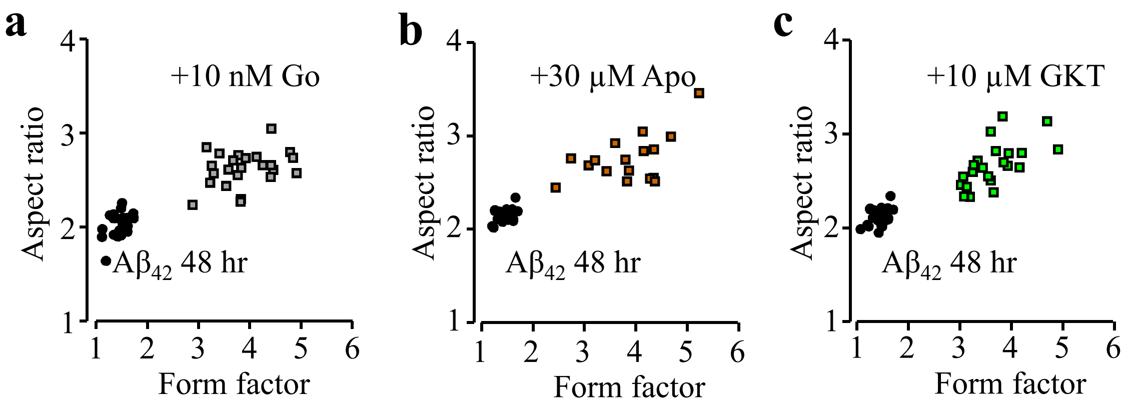

Supplementary Fig. 12

a

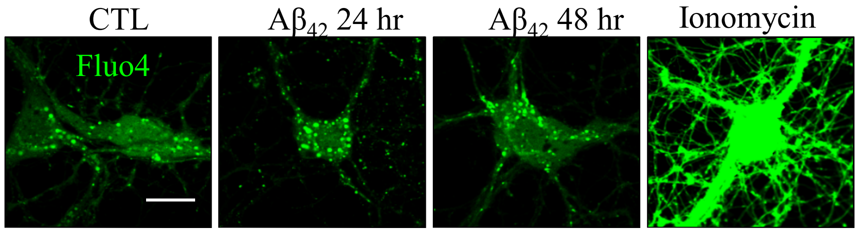

b

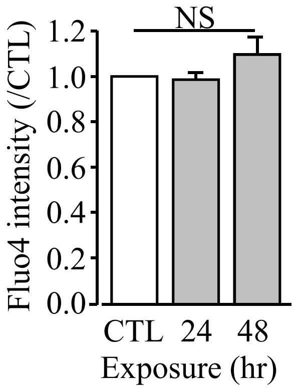

Supplement: Supplementary file 1 — supplementary data [file 41419_2018_270_MOESM1_ESM.pdf]
